# Putative purine nucleoside interacting residues in the malaria parasite purine uptake transporter PfENT1 are critical for transporter function

**DOI:** 10.1371/journal.pone.0293923

**Published:** 2023-12-19

**Authors:** Criselda Dillague, Myles H. Akabas

**Affiliations:** 1 Department of Neuroscience, Albert Einstein College of Medicine, Bronx, New York, United States of America; 2 Department of Medicine, Albert Einstein College of Medicine, Bronx, New York, United States of America; Zhejiang University College of Life Sciences, CHINA

## Abstract

Malaria remains a major public health threat for billions of people worldwide. Infection with obligate intracellular, unicellular parasites from the genus *Plasmodium* causes malaria. *Plasmodium falciparum* causes the deadliest form of human malaria. *Plasmodium* parasites are purine auxotrophic. They rely on purine import from the host red blood cell cytoplasm via equilibrative nucleoside transporters to supply substrates to the purine salvage pathway. We previously developed a high throughput screening assay to identify inhibitors of the *P*. *falciparum* Equilibrative Nucleoside Transporter Type 1 (PfENT1). Screening a small molecule library identified PfENT1 inhibitors that blocked proliferation of *P*. *falciparum* parasites in *in vitro* culture. The goal of the current work was to validate a high-resolution model of PfENT1 predicted by the AlphaFold protein structure prediction program. We superimposed the predicted PfENT1 structure on the human homologue structure, hENT1, and developed a structure-based sequence alignment. We mutated the residues in PfENT1 aligned with and flanking the residues in hENT1 that interact with the purine analog, nitrobenzylthioinosine (NBMPR). Mutation of the PfENT1 residues Q135, D287, and R291 that are predicted to form hydrogen bonds to purine nucleosides eliminated purine and pyrimidine transport function in various yeast-based growth and radiolabeled substrate uptake assays. Mutation of two flanking residues, W53 and S290, also resulted in inactive protein. Mutation of L50 that forms hydrophobic interactions with the purine nucleobase reduced transport function. Based on our results the AlphaFold predicted structure for PfENT1 may be useful in guiding medicinal chemistry efforts to improve the potency of our PfENT1 inhibitors.

## Introduction

Malaria remains a worldwide public health problem. In 2021 the World Health Organization (WHO) reported over 247 million cases of malaria and more than 600,000 deaths worldwide [1]. Malaria is caused by infection with a *Plasmodium* parasite. Five species are known to cause malaria in humans. *Plasmodium falciparum* is responsible for the highest patient mortality rate, with most of these fatalities occurring in children and pregnant women [2]. One way to alleviate the global malaria burden is through the development of vaccines. Recently, the WHO approved a malaria vaccine for children from 0–5 years of age, who are one of the populations most susceptible to infection and death in malaria-endemic countries. Unfortunately, the vaccine loses efficacy over a few years [3, 4]. Because protection by the vaccine wears off, additional tools are needed to ameliorate the impact of malaria infection. These tools include efficacious antimalarial drugs. Artemisinin-based combination therapies (ACTs) are the current first-line therapy for the treatment of malaria. However, in recent years, the rise and spread of artemisinin resistance and the presence of resistance to common partner drugs in ACT-resistant parasites highlight the need for development of novel antimalarial drugs [5–8].

One promising antimalarial drug target is the *P*. *falciparum* Equilibrative Nucleoside Transporter 1 (PfENT1). *Plasmodium* parasites are purine auxotrophic; they lack pathways for *de novo* purine synthesis. *Plasmodium* parasites import purines from the host red blood cell (RBC) and utilize the purine salvage pathway to synthesize adenylate and guanylate nucleotides [9]. Equilibrative nucleoside transporters (ENTs) are the primary pathway for purine import. The *P*. *falciparum* genome has four putative ENTs, with ENT type 1 (PfENT1) being the major purine importer. A knock-out of PfENT1 (*pfent1Δ*) resulted in conditional lethality to parasites [10, 11], showing its importance for asexual parasite proliferation. Moreover, a recent study using transposon saturation mutagenesis classified *pfent1* as an essential gene during the asexual life cycle [12]. Because PfENT1 is essential for asexual blood stage parasite proliferation, we hypothesize that PfENT1 could be a promising antimalarial drug target.

Previously, to identify potential PfENT1 inhibitors, we collaborated with GlaxoSmithKline (GSK) and utilized our previously established yeast-based, high-throughput screen (HTS) [13] to screen their 1.8 million compound library [14]. From the HTS, GSK provided us with six compounds, named GSK-1 to GSK-6, as potential starting points for drug development based on potency, ligand efficiency, physiochemical properties, and their structure-activity relationship (SAR) [14]. We extensively characterized GSK-1 to GSK-6 using orthogonal yeast-based assays and demonstrated that these GSK compounds inhibited the proliferation of antimalarial drug-resistant *P*. *falciparum* strains (Dd2, HB3, 7G8, Cam3.II artemisinin resistant) in *in vitro* culture. Additionally, we demonstrated that these GSK compounds inhibited PfENT1 with half-maximal inhibitory concentrations (IC_50_ value) in the micromolar range using inhibition of radiolabeled substrate uptake assays [14]. Our previous results established that the GSK compounds inhibit PfENT1. However, when we embarked on this study, the location of the GSK compound binding site(s) with PfENT1 were unknown.

A barrier to identifying the location of the GSK compounds’ binding site(s) on PfENT1 was the lack of a high-resolution structure of PfENT1. A high-resolution structure of the human homolog of PfENT1, human equilibrative nucleoside transporter 1 (hENT1) had been reported with the purine analog inhibitor, nitrobenzylthioinosine (NBMPR), bound [15]. The hENT1 residues Gln158, Asp341, and Arg345 formed hydrogen bonds to the NBMPR nucleobase and ribose ring. Leu26 formed a hydrophobic interaction with the purine ring [15]. The low degree of amino acid sequence identify, about 17%, made it difficult to create a reliable sequence alignment between PfENT1 and hENT1. Without that it was difficult to generate a homology model. In 2021, the AlphaFold team predicted the protein structures for most *P*. *falciparum* proteins, including PfENT1 [16]. We used the Chimera Matchmaker function to superimpose the AlphaFold predicted PfENT1 structure and the hENT1 structure and create a structure-based amino acid sequence alignment. We generated mutations in the PfENT1 residues predicted to align with the hENT1 purine binding residues and also mutated the residues flanking the putative binding site residues. We expressed the mutants in our yeast-based expression system and assessed the functional impact of the mutations. The results are consistent with the importance of the purine nucleoside interacting residues for PfENT1 function. The experimental support for this structure confirms it as a useful tool for the characterization of the binding site(s) for the PfENT1 inhibitory compounds and may facilitate further medicinal chemistry efforts to improve the GSK compounds’ potency and efficacy.

While this manuscript was in preparation, a paper was published that reported high resolution cryo-electron microscopy structures of PfENT1 with inosine and with GSK-4 bound in the transport pathway [17]. They mutated to alanine five of the 16 residues mutated to cysteine in this work and functionally characterized the mutants by inosine binding isothermal calorimetry and transport. At four of the five positions, the observed effects on inosine transport are consistent with the results of this paper. We discuss potential explanations for the differences observed at the other position in the Results and Discussion section.

## Materials and methods

### Generating model and alignment using Chimera

**Fig 1:**
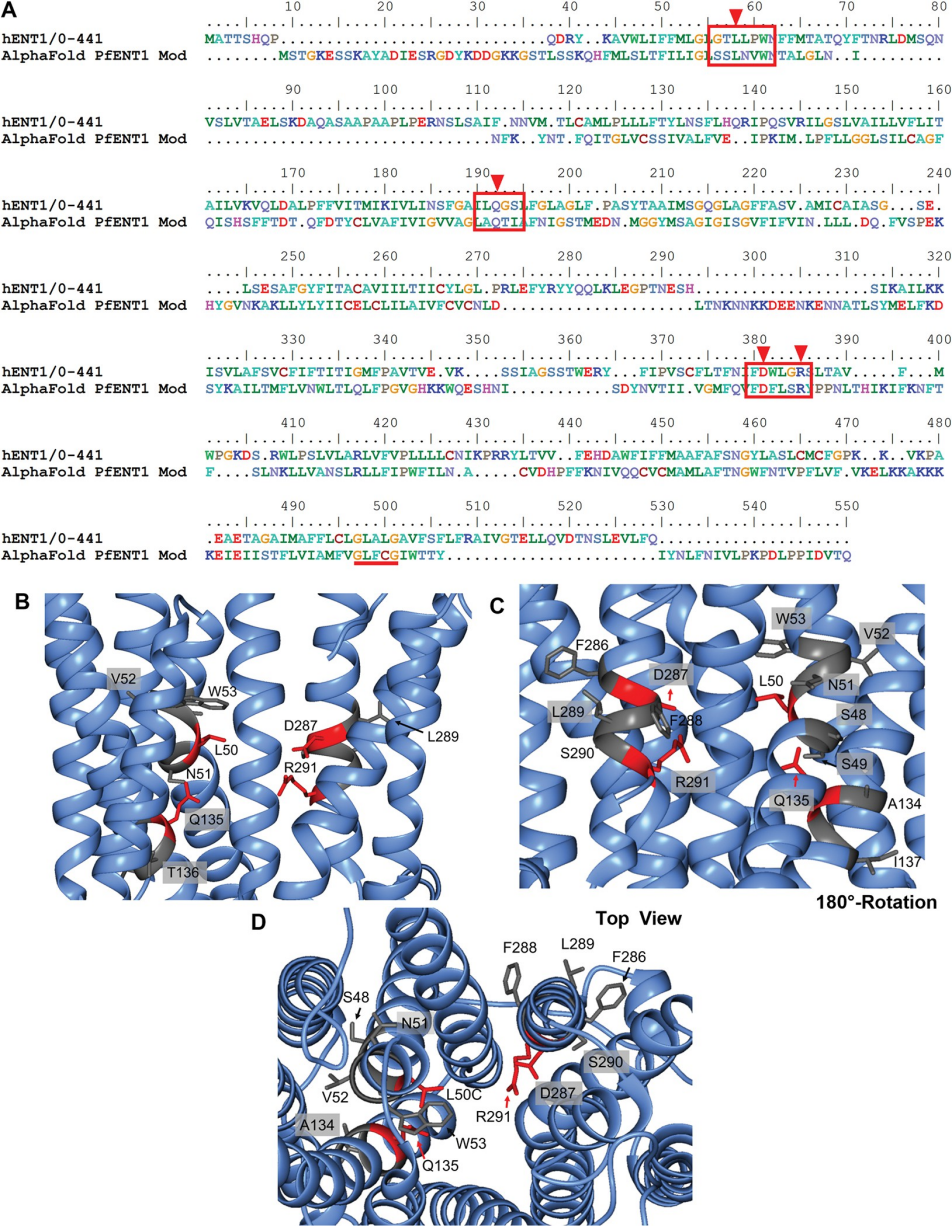
Structure-based sequence alignment of hENT1 and PfENT1 and AlphaFold predicted structure of the PfENT1 residues predicted to align with purine interacting residues in the hENT1 crystal structure with NBMPR bound. (A) Alignment of PfENT1 (top sequence) and hENT1 (bottom sequence) generated through the “Match -> Align” function in Chimera after superimposing both structures. The highly conserved GXXXG motif in TM11 is underlined in red. The PfENT1 residues aligned with the purine interacting residues in hENT1 are highlighted with red arrows. They correspond to L50, Q135, D287 and R291 in PfENT1. Residues in the red box identify residues flanking the purine interacting residues that were mutated in this study. (B) AlphaFold model of the PfENT1 transmembrane region surrounding putative purine binding residues in an outward open conformation. Residues mutated in this study in and around the putative purine binding site are shown in stick representation. Those in red are aligned with the purine interacting residues in the hENT1 structure. Those in gray are the flanking residues. (C) 180-degree rotation of the view in panel B. (D) The view from the extracellular side looking into the transporter.

The Protein Data Bank (PDB) files of PfENT1 generated by AlphaFold (AF-Q8IDM6-F1) [16] and the hENT1 structure with NBMPR bound (6ob6) [15] were obtained. Both files were then uploaded into the Chimera [18] “Match -> Align” function to generate a structural alignment and a resultant amino acid sequence alignment. This sequence alignment was used to identify the PfENT1 residues that aligned with the hENT1 residues L26, Q158, D341, and R345. Molecular models in Fig 1 were generated with Chimera.

### Site directed mutagenesis and generation of yeast mutants

Site directed mutagenesis was performed on the yeast codon optimized PfENT1 encoding gene in the pCM189 episomal expression plasmid that we developed previously [13]. We used oligonucleotides designed by the NEBBaseChanger program (https://nebasechanger.neb.com/) to introduce the point mutations in the PfENT1 gene. Oligonucleotides were obtained from GENEWIZ (https://www.genewiz.com/). The Q5® Site-Directed Mutagenesis Kit from New England Biolabs (NEB Cat. #E0554S) was used. Plasmids containing the mutations were transformed into DH5α Competent Cells (NEB Cat. #C2987H) and grown on solid LB media containing ampicillin as the selectable marker. Successfully transformed bacteria were grown and the PfENT1 putative mutant plasmids were purified using the NEB Monarch® Plasmid Miniprep Kit (NEB Cat. #T1010S). All mutations were confirmed by DNA sequencing of the entire coding region.

Plasmids with correct mutations were transformed into purine auxotrophic yeast (genotype *MAT*a, *his3*Δ1, *leu2*Δ0, *met15*Δ0, *ura3*Δ0, *fui1*Δ::kanMX4, *ade2*Δ::hphNT1) that we described previously [13] using the ZYMO Frozen-EZ Yeast Transformation II Kit (ZYMO Cat. #T2001). Purine auxotrophic yeast were initially grown in YPD media containing 1% (w/v) yeast extract, 2% (w/v) peptone, and 2% (w/v) dextrose. Following transformation, yeast were plated on 3% agar synthetic defined media (SDM) plates. Individual colonies were picked and expanded in liquid SDM containing 1 mM adenine. The SDM used for these yeast contained 0.67% (w/v) yeast nitrogen base (US Biologicals Cat. #Y2025), 0.2% (w/v) drop-out mix (US Biologicals Cat. #D9517-04A; without histidine, leucine, adenine), 2% (w/v) dextrose (Sigma Cat. #G7021), 0.004% L-Histidine (Sigma Cat #H8000), 0.004% L-Leucine (Sigma Cat. #L8000), 1 mM adenine (Sigma Cat. #A9251). Lastly, the SDM contained hygromycin B as the selection marker for yeast lacking ADE2. Successful transformants were frozen down and grown as needed for the experiments.

### 24-hour growth assays

The Bioscreen C (Model #: 61400PRO) machine was used to measure proliferation of yeast expressing either wild type or mutant PfENT1 in 24-h assays. Yeast strains were grown overnight in SDM media plus 1 mM adenine. Yeast were spun down and resuspended in sterile water. Yeast were added into wells of honeycomb plates specific for the Bioscreen C (Fisher Scientific Cat No. #NC9976780) to achieve a final OD_600_ of 0.05 in the total volume of 150 μl per well of yeast and media. With the yeast added, each well contained a final concentration of 1x SDM media with either 1 mM or 2 mM adenosine, and 0.1% NP40 (Sigma-Aldrich Cat No. #492018) to prevent clumping for an initial OD_600_ of 0.05. Each trial contained technical triplicates of every sample. The honeycomb plates were placed in the Bioscreen C where they were shaken continuously on high for 24-h at 30°C. OD_600_ was measured every 15 min. A growth curve was generated, and the doubling time was calculated for each strain that showed exponential growth.

### Radiolabeled uptake assays

The radiolabeled uptake assay measures the ability of an unlabeled purine or pyrimidine substrate to inhibit the uptake of radiolabeled substrate into yeast via PfENT1 as previously described [14, 19]. Yeast strains containing PfENT1 (mutant or WT) were grown overnight in SDM media to a final OD_600_ between 0.2 and 0.5. Purine auxotrophic yeast used as a negative control were grown in YPD media. Yeast were spun down and washed three times with 15 mL phosphate buffered saline (PBS; 137 mM NaCl, 2.7 mM KCl, 10 mM KH_2_PO_4_, 10 mM Na_2_HPO_4_, pH 7.4). The final volume of yeast in PBS varied but it was diluted to a final OD_600_ = 0.2. 100 μL of PfENT1-expressing or purine auxotrophic yeast were added into each well that contained 50 μL of 200 nM [^3^H]adenosine or [^3^H]uridine, and 50 μL of the serially diluted unlabeled uridine or adenosine. For the dilution series, unlabeled purine/pyrimidine (Fischer Scientific Cat. #12-565-501) with a starting concentration of 25 mM was serially diluted 1:3 in PBS for a final concentration range of 3.28 nM to 6.25 mM. Yeast were incubated with radioactive substrate and the unlabeled purine/pyrimidine for 15 min. Cold uridine was competed against [^3^H]adenosine (Moravek Cat. #MT793), cold adenosine was competed against while [^3^H]uridine (Moravek Cat. #MT799M). This ensured that competition only occurred at the transporter and not at cytoplasmic metabolic enzymes, because those are different for purines and pyrimidines. For determination of minimal and maximal uptake, yeast strains were incubated without radioactivity or with radioactivity but no cold substrate competition, respectively. After 15 min, the yeast were harvested onto glass fiber filter mats (Perkin-Elmer Cat. #1450–421) using the TomTec Harvester (Model #96-3-469). Mats were allowed to air dry and sealed in sample bags with 5 mL of BetaPlate Scint scintillant cocktail (Perkin Elmer Cat. #1205–4401). The amount of radioactivity in each well was measured by liquid scintillation counting using a 1450 MicroBeta TriLux (Perkin Elmer). The counts per minute (cpm) were recorded and technical triplicates were averaged. IC_50_ values were calculated, and statistical analysis was performed using GraphPad/Prism9 software. Each experiment was repeated at least three times on separate days.

### 5-Fluorouridine (5-FUrd) toxicity assay

5-FUrd toxicity was measured in a subset of the PfENT1 mutants. Mutants were grown overnight in SDM with 1 mM adenine. Meanwhile, purine auxotrophic yeast were grown in YPD overnight. Yeast strains were spun down and resuspended in sterile water then added to the honeycomb plates with SDM media with NP40 (final concentration of 0.1%) containing either no adenine, 500 μM adenine, or 500 μM adenine plus 125 μM 5-FUrd (Fisher Scientific Cat. #AAJ6208303) with an initial OD_600_ = 0.05 and a final volume of 150 μL. Typically, 10 μL of yeast (final concentration of resuspended yeast OD_600_ = around 0.8) were added to 140 μL of media. Each trial contained triplicates. The 100-well honeycomb plate was placed in the Bioscreen C for 24-h under continuous high shaking at 30°C. The endpoint OD_600_ was measured at 24 h. Each experiment was repeated at least three times on separate days.

### Computational and statistical analysis

Statistical significance was calculated using one-way analysis of variance (ANOVA) with GraphPad/Prism9 software. IC_50_ values were calculated using the non-linear regression dose-response inhibition log(inhibitor) vs normalized response program. Cpm data was background subtracted by the cpm in yeast expressing WT PfENT1 in cold purine/pyrimidine. Data was normalized to the highest value for each mutant.

For calculating the doubling time, the formula used was:

$$Dt=\frac{ln(2)}{k}where\;k=the\;growth\;rate$$

The k was calculated in Excel using the OD_600_ values in the growth curve that represented exponential growth (OD_600_ between 0.2 and 0.6). Alternatively, this formula can also be used [20]:

$$Dt=\frac{ln(2)}{\left(\frac{\left[ln(OD_{2})-\ln\left(OD_{1}\right)\right]}{t_{2}-t_{1}}\right)}$$

## Results and discussion

### Identification and mutation of putative purine binding residues in PfENT1

We sought to identify the putative purine binding residues in PfENT1. As a starting point we used the high-resolution structure of a human PfENT1 homolog, hENT1, co-crystalized with a purine analog transport inhibitor, NBMPR. In the hENT1 structure, the residues Q158, D341, and R345 were identified as forming hydrogen bonds to the purine nucleoside and therefore are likely to be important determinants of purine binding [15]. L26 forms a hydrophobic interaction with the purine moiety [15]. We hypothesized that the PfENT1 residues aligned with these hENT1 residues would also play a role in purine binding.

The low sequence identity between hENT1 and PfENT1 made it difficult to generate a reliable sequence alignment. Amino acid sequence-based alignments between the hENT1 and PfENT1 sequences resulted in no more than 17% sequence identity and failed to align the highly conserved GXXXG motif in TM11 that is found in all equilibrative nucleoside transporters [21]. Therefore, prediction of which PfENT1 residues aligned with the purine binding residues in hENT1 was uncertain. To overcome this obstacle, we started with the AlphaFold predicted PfENT1 protein structure published in 2021 [16, 22]. We used Chimera’s MatchMaker function to construct a structure-based alignment of the hENT1 structure and the predicted PfENT1 structure [18]. This generated an amino acid sequence alignment where the GXXXG motifs aligned and where the amino acids at the purine interacting positions in hENT1 were conserved in PfENT1 (Fig 1A). The PfENT1 residues L50, Q135, D287, and R291 aligned with the hENT1 purine interacting residues.

To determine whether the PfENT1 residues L50, Q135, D287, and R291 are important for purine binding, we mutated each of them and the surrounding residues to cysteine, individually. We mutated the surrounding residues to probe the local environment and the accuracy of the sequence alignment. The surrounding residues included S48, S49, N51, V52, W53, A134, T136, I137, F286, F288, L289, and S290 (Fig 1). We chose to mutate them to cysteine to facilitate potential cysteine accessibility studies in subsequent experiments [23].

We generated 16 individual PfENT1 cysteine-substitution mutants in a yeast codon-optimized *pfent1* gene cassette in the pCM189 yeast episomal expression plasmid [13]. Following transformation into our previously established purine auxotrophic yeast background (*ade2Δ*, *fui1Δ*) the yeast express the corresponding PfENT1 mutant [13, 14]. This yeast strain lacks the ADE2 enzyme, essential in the *de novo* purine biosynthesis pathway [24], and the endogenous FUI1 uridine transporter. This yeast strain requires an exogenous purine source. It grows well on purine nucleobases because yeast have endogenous nucleobase transporters. However, yeast lack purine nucleoside transporters, so with adenosine as the sole purine source, PfENT1 is the only pathway for adenosine to enter the yeast. Lacking the FUI1 transporter makes the yeast resistant to the cytotoxic effects of 5-flurouridine (5-FUrd).

### Effect of PfENT1 mutations on yeast proliferation in media containing adenosine as the sole purine source

**Fig 2:**
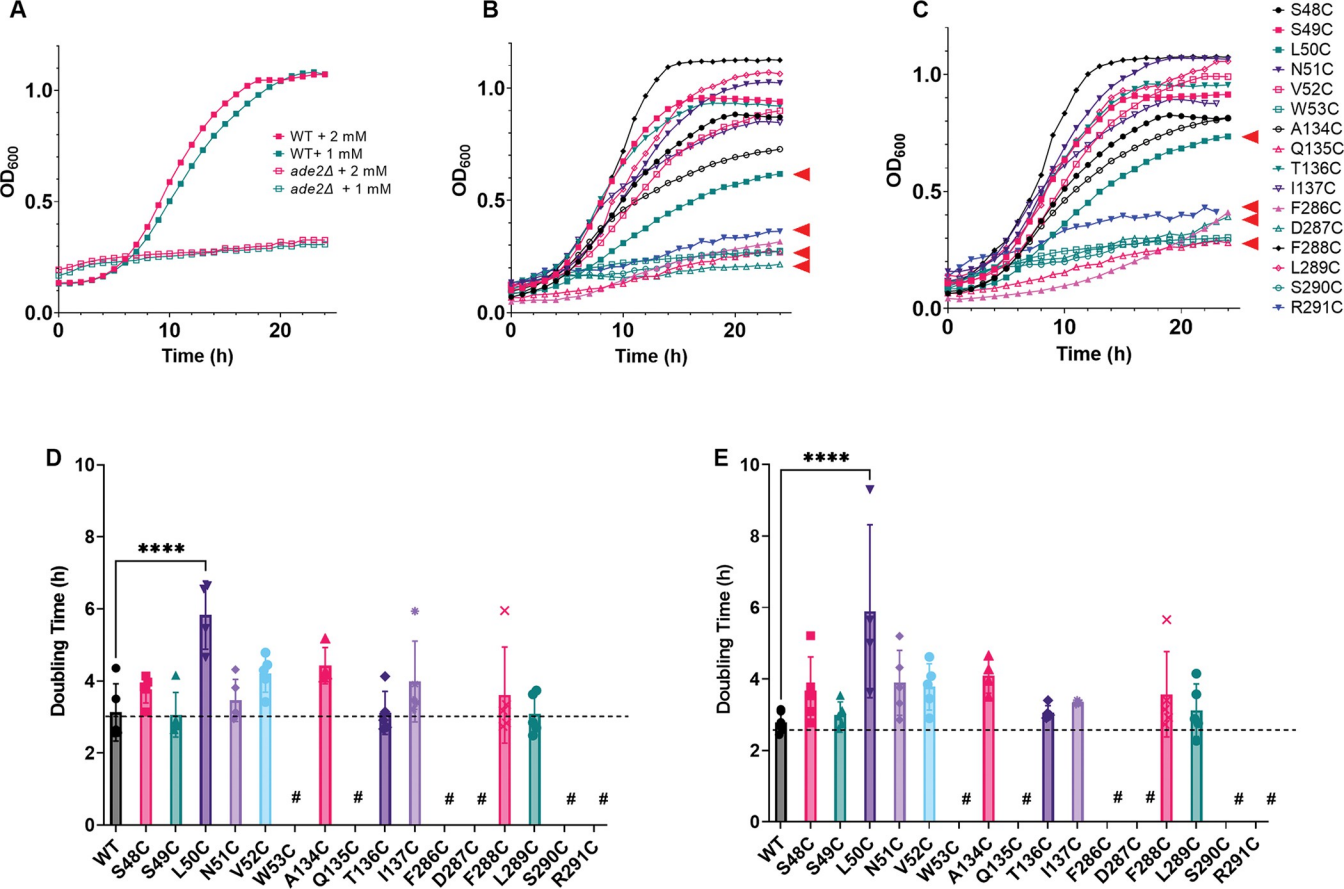
Yeast expressing PfENT1 containing mutations to putative purine binding site residues have growth defects in 24 hour adenosine-dependent growth assay. For this assay, adenosine is the only purine source for the yeast (n = 5). (A) Growth of purine auxotrophic yeast (*ade2Δ/fui1*Δ) that do not (open squares) or do (closed squares) express WT PfENT1 in 1 mM or 2 mM adenosine. Yeast expressing PfENT1 proliferated in both adenosine concentrations while purine auxotrophic yeast not expressing PfENT1 did not proliferate. (B & C) Twenty-four-hour growth curves for yeast expressing various PfENT1 mutants in (B) 1 mM adenosine or (C) 2 mM adenosine. The yeast expressing PfENT1 containing mutations to putative purine binding site residues are highlighted with red arrows. The yeast expressing PfENT1 with W53C, Q135C, D287C, S290C, and R291C did not proliferate at either adenosine concentration. Meanwhile yeast expressing PfENT1 with the L50C or the F286C mutation did proliferate but not as well as yeast expressing WT PfENT1. (D) The doubling times of the strains grown in 1 mM adenosine. (E) The doubling times of the strains grown in 2 mM adenosine. Doubling times are depicted with the mean, SEM, and the points representing the values from five independent trials. Yeast expressing L50C PfENT1 have a significantly longer doubling time compared to WT PfENT1 expressing yeast by one-way ANOVA (****, p<0.0001). The # symbol indicates that no doubling time was calculated either because these mutants did not grow or grew too slowly to determine a doubling time in the 24 h assay.

We characterized the growth rate of yeast expressing each of the PfENT1 mutants in a 24 h adenosine-dependent proliferation assay. When adenosine is the sole purine source, the PfENT1-mutant expressing yeast strains can only proliferate if the expressed PfENT1 mutant is functional (Fig 2A). If a mutation affects PfENT1 function or plasma membrane expression level, we expect it will affect yeast proliferation rate in media with adenosine as the sole purine source. Previously, our laboratory used 1 mM adenosine as the standard concentration for overnight cultures. For the present experiments, we also tested proliferation rates in 2 mM and 10 mM adenosine. Fig 2A illustrates that the purine auxotrophic yeast background strain does not proliferate in media with adenosine as the sole purine source. However, when wild type (WT) PfENT1 is expressed in this background strain the yeast proliferated at similar rates in 1 and 2 mM adenosine and displayed similar sigmoidal growth curves. In media containing 10 mM adenosine the PfENT1-expressing yeast grew faster and plateaued at a higher OD_600_ than those grown in 1 or 2 mM adenosine (S1 Fig). However, in 10 mM adenosine there was some proliferation of the purine auxotrophic yeast background (*ade2Δ*, *fui1Δ*) strain (S1 Fig). We do not know whether this was due to adenine contamination of the adenosine or expression of nucleosidases activity from the yeast that was able to provide sufficient adenine for cell proliferation. Therefore, for the cysteine-substitution mutants, we only tested the proliferation rates in media containing 1 or 2 mM adenosine.

We performed 24 h growth assays in media containing 1 or 2 mM adenosine with yeast expressing each of the cysteine-substitution mutations in and around the putative PfENT1 purine binding residues. Yeast expressing mutations at three of the four putative binding site residues, Q135C, D287C, and R291C, resembled the growth curves of the purine auxotrophic yeast. (Fig 2 and Table 1). We had hypothesized that the Q135C, D287C, and R291C PfENT1 mutants would have growth defects as these residues were predicted to form hydrogen bonds to the purine nucleoside. Given the absence of function, we cannot exclude the possibility that these mutations result in a protein folding defect that results in the absence of properly folded PfENT1 in the plasma membrane leading or that the mutations prevent the protein from undergoing the conformational changes necessary for transport.

**Table 1:** Summary of the results from all assays for yeast expressing the indicated mutant or WT, and the purine auxotrophic background strain.

	Avg. Doubling time (h)		5-FUrd assay	Average IC_50_ (μM)		Mutant/WT Ratio		
	Adenosine growth assay							
Strain	1 mM	2 mM		Adenosine	Uridine	Adenosine	Uridine	TM Location
ade2Δ	ng	ng	growth-non-functional	no uptake	no uptake	-	-	-
WT	3.13±0.36	3.72±0.21	killed-functional	101.1±60	997±90	-	-	-
S48C	3.77±0.17	3.68±0.42	nt	64.4±4.5	801±46	0.64	0.8	1
S49C	3.06±0.28	2.99±0.16	nt	87.4±9.8	1060±30	0.86	1.06	1
L50C	5.83±0.47*	7.16±1.21*	killed-functional	no uptake	no uptake	-	-	1
N51C	3.46±0.26	3.89±0.41	killed-functional	74.9 ± 2.8	30.9 ± 1.0*	0.73	0.031	1
V52C	4.20±0.23	3.78±0.29	nt	215.6±43.0	2822±1435*	2.13	2.83	1
W53C	ng	ng	growth-non-functional*	no uptake	no uptake	-	-	1
A134C	4.42±0.25	4.09±0.24	nt	79.5±9.4	1019±207	0.79	1.02	4
Q135C	ng	ng	growth-non-functional*	no uptake	no uptake	-	-	4
T136C	3.12±0.27	3.06±0.09	nt	60.9±10.9	786±87	0.6	0.79	4
I137C	3.99±0.50	3.5±0.16	nt	62.1±4.3	904±21	0.61	0.91	4
F286C	slow growth	slow growth	killed-functional	273.4±97.2	1210±139	2.7	1.21	8
D287C	ng	ng	growth-non-functional*	no uptake	no uptake	-	-	8
F288C	3.61±0.60	3.57±0.53	nt	60.1±0.4	850±138	0.59	0.85	8
L289C	3.08±0.25	3.12±0.33	nt	97.7±15.2	905±164	0.97	0.91	8
S290C	ng	ng	growth-non-functional*	no uptake	no uptake	-	-	8
R291C	ng	ng	growth-non-functional*	no uptake	no uptake	-	-	8

The putative purine binding residues are highlighted in yellow. A minimum of three trials were performed per strain per assay (n≥3). Summary of the average doubling time and SEM is shown for each yeast strain. Mutant strains that did not grow are indicated by (ng, no growth). For the F286C expressing yeast, there was slow growth but it was not sufficient to calculate a doubling time during the 24 h growth assay. Results of the 5-FUrd assay are indicated as “killed-functional”, “growth-non-functional”, or not tested (nt). For the radioactive substrate uptake assays average IC_50_ values and SEM are shown. The Mutant/WT ratio of the IC_50_ values are shown, as well as the transmembrane segment location of each mutation (TM Location). Asterisk (*) indicates significantly different than WT by one way ANOVA (see figure legends for p values).

In contrast to the other three predicted purine binding residues, yeast expressing the L50C mutant did grow, albeit at a slower rate than yeast expressing WT PfENT1. In the media containing 1 and 2 mM adenosine, L50C-expressing yeast exhibit increased growth compared to the purine auxotrophic background control. However, it appeared that L50C growth was impaired because they grew to a lower plateau OD_600_ compared to yeast expressing WT PfENT1 (Fig 2B and 2C). In both 1 mM and 2 mM adenosine, the L50C mutant yeast doubling times were significantly increased, 1.9-fold compared to WT PfENT1 control (5.8 h vs. 3.1 h p<0.0001; and 7.2 h vs. 3.7 h p<0.0001, respectively) (Fig 2D and 2E, Table 1). Using a one-way ANOVA, the L50C mutant yeast doubling times were significantly longer than WT. These results show that the L50C mutation impacts PfENT1 transporter function or expression but is not lethal. This may be due to the fact that hENT1 L26, aligned with L50, has hydrophobic interactions with the purine ring [15]. In the PfENT1 cryo-EM structure, L50 is close to but does not directly interact with inosine [17]. This difference from the hENT1 structure might be due to the fact that the hENT1 structure is open outward and the PfENT1 structure is open inward. In contrast, the other three purine binding residues form H-bond interactions with either the purine ring, Q158, or the ribosyl hydroxyls, D341 and R345 in the hENT1 structure [15] and with inosine in the PfENT1 structure [17].

Yeast expressing most of the mutations in residues flanking the binding site residues grew in a manner similar to yeast expressing WT PfENT1 (Fig 2 and Table 1). The growth and doubling time were not significantly different than WT, except for the W53C, F286C, and S290C mutants (Fig 2B–2E). Yeast expressing F286C PfENT1 grew significantly more than the purine auxotrophic background strain (Fig 2) but did not grow enough in the 24 h assay to reach saturation or to calculate a doubling time.

In contrast, the growth of the W53C and S290C mutants resembled the growth curves of the purine auxotrophic yeast (Fig 2). We were unable to calculate the doubling time for yeast expressing these mutants (Fig 2D and 2E, Table 1). This suggests that for these mutants, there was no functional adenosine transporter in the yeast cell plasma membrane. We did not expect that the mutations W53C and S290C would have such a dramatic impact on PfENT1 function because in the hENT1 structure, which is outward facing, the aligned residues did not directly interact with the NBMPR purine nucleobase or ribose moieties [15]. In contrast, in the PfENT1 structure, which is inward open with a nanobody bound in the cytoplasmic entrance to the permeation pathway, W53 interacts with the hydroxyl on the ribose 5’ carbon residue [17]. They also report that the tryptophan limits the rotation of the inosine in the binding site. Thus, replacement of the W53 with a cysteine may alter these interactions an prevent substrate binding and/or transport. Mutation of these residues may also alter the orientation of the adjacent binding site residues preventing them from interacting with purine substrates. Alternatively, these mutations may have affected the conformational changes required for purine transport or protein folding resulting in a failure of the resultant protein to traffic to the plasma membrane. We cannot distinguish between these potential interpretations.

### 5-fluorouridine (5FUrd) cytotoxicity assay confirmed the functional status of the mutants with altered function in the adenosine-dependent growth assay

**Fig 3:**
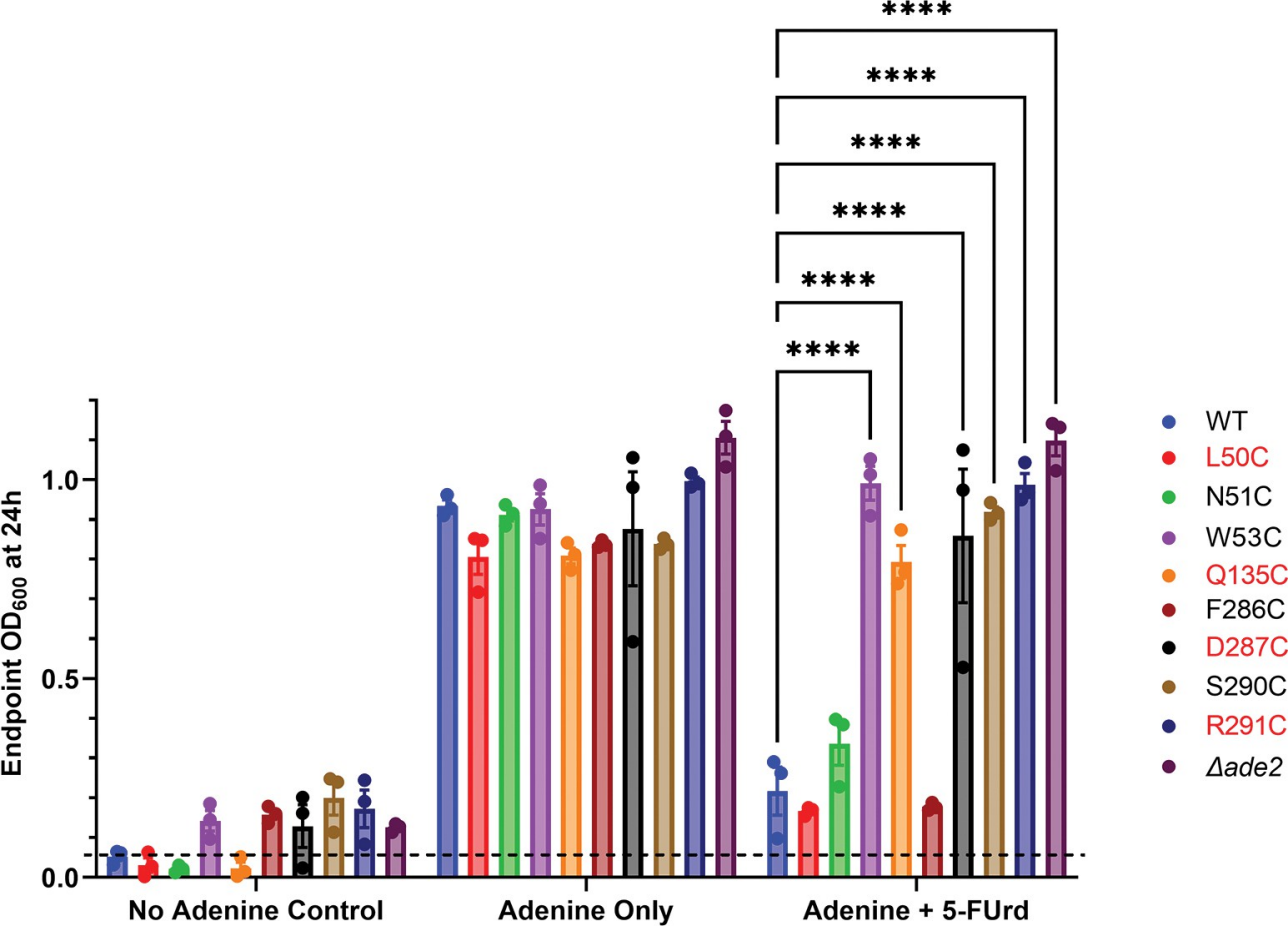
Effect of growth in the presence of 5-fluorouridine (5-FUrd) on the proliferation of WT and selected PfENT1 mutants expressed in purine auxotrophic yeast. Yeast express an endogenous nucleobase transporter and can grow in the presence of adenine as the sole purine source. Yeast proliferation was assessed by OD_600_ measured after 24 h in culture. Each trial was performed in triplicates. Each data point represents the average of the triplicate in each trial. Mean OD_600_, SEM, and individual experiments are shown for each condition (n = 3). No adenine control (left) shows that none of the yeast proliferated in the absence of a purine source. In the presence of adenine (middle), all of the yeast strains proliferated. When grown in the presence of adenine and 125 μM 5-FUrd (right), strains expressing WT or functional PfENT1 mutants did not proliferate, but those with non-functional PfENT1 mutants did proliferate because there was no entry pathway for 5-FUrd. Significant differences were determined by one-way ANOVA compared to WT (****, *p<0*.*0001*).

We used our orthogonal 5-FUrd cytotoxicity assay to confirm the functional status of the mutants that displayed altered function in the adenosine-dependent growth assay. Wild type yeast express a uridine transporter, *FUI1*, and are killed by growth in the presence of the cytotoxic uridine analog 5-FUrd [13, 25]. Our yeast background lacks this endogenous *FUI1* transporter (*fui1Δ*) and thus can grow in the presence of 5-FUrd. However, PfENT1 transports uridine and 5-FUrd [26]. When functional PfENT1 is expressed in the purine auxotrophic (*ade2*Δ, *fui1*Δ) background they re-gain the ability to import 5-FUrd and are killed by growth in the presence of 5-FUrd. If PfENT1 mutations result in a nonfunctional transporter, then 5-FUrd will not be imported, and the yeast will proliferate in the presence of adenine as the sole purine source. If the transporter is functional, 5-FUrd will be imported, resulting in yeast death when grown in the presence of 5-FUrd. Neither WT nor mutant expressing yeast grew in the absence of adenine (Fig 3, left panel). All grew in the presence of 500 μM adenine as the sole purine source because the yeast express endogenous nucleobase transporters (Fig 3, middle panel). In the presence of adenine and 125 μM 5-FUrd, yeast expressing WT, L50C, N51C, and F286C did not grow indicating they expressed functional PfENT1. In contrast, yeast expressing W53C, Q135C, D287C, S290C, and R291C did grow, indicating that PfENT1 was not functional for these mutants (Fig 3, right panel). These results are consistent with the results of the adenosine-dependent growth assay and confirm that even though the F286C mutant grew slowly, it was functional in both assays.

### Effect of PfENT1 mutations on affinity for transported substrates

**Fig 4:**
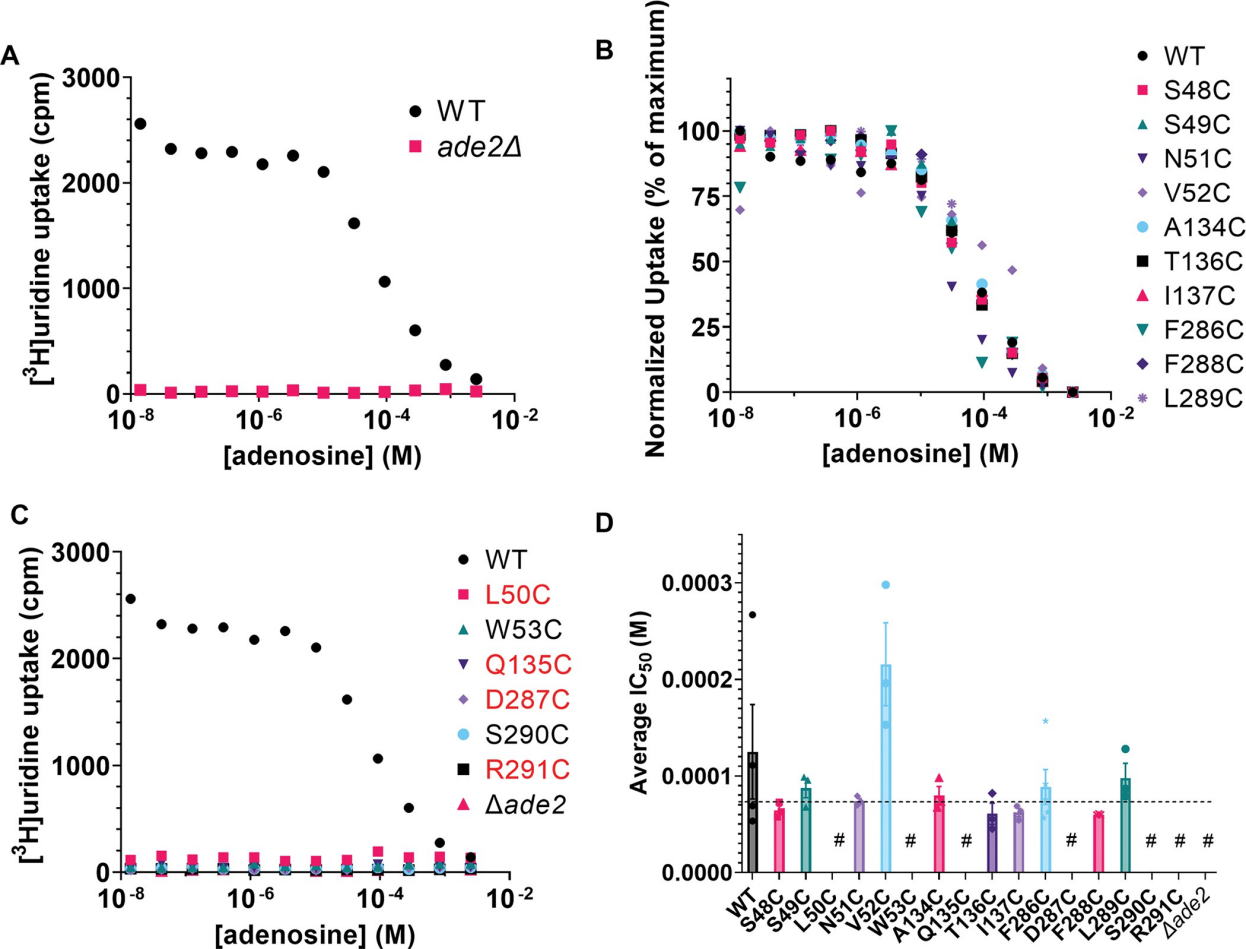
Assessment of WT and mutant PfENT1 function by 15 min [^3^H]uridine uptake assay. (A) Representative experiments showing adenosine competition with [^3^H]uridine uptake by the background purine auxotrophic yeast strain (*ade2*Δ*/fui1*Δ) (brown squares) and for that strain expressing WT PfENT1 (blue circles). The purine auxotrophic background strain did not import [^3^H]uridine while yeast expressing WT PfENT1 imported [^3^H]uridine and displayed concentration-dependent inhibition of uptake by cold adenosine. (B) Normalized [^3^H]uridine uptake competition with cold adenosine for all of the mutants that were functional in the adenosine-dependent proliferation assays, except L50C. These showed [^3^H]uridine uptake and concentration-dependent inhibition of [^3^H]uridine uptake by adenosine. (C) Representative experiments showing lack of [^3^H]uridine uptake for those PfENT1 mutants that were not functional in the adenosine-dependent proliferation assay and for L50C. Uptake by WT PfENT1 expressing yeast is shown as a positive control (blue circles). (D) Calculated IC_50_ values for adenosine inhibition of [^3^H]uridine uptake in the functional mutants and WT. Mean, SEM, and individual data points are shown (n≥3). Statistical significance was calculated by one-way ANOVA, none were significantly different compared to WT. The # symbol indicates mutants that did not import [^3^H]uridine.

**Fig 5:**
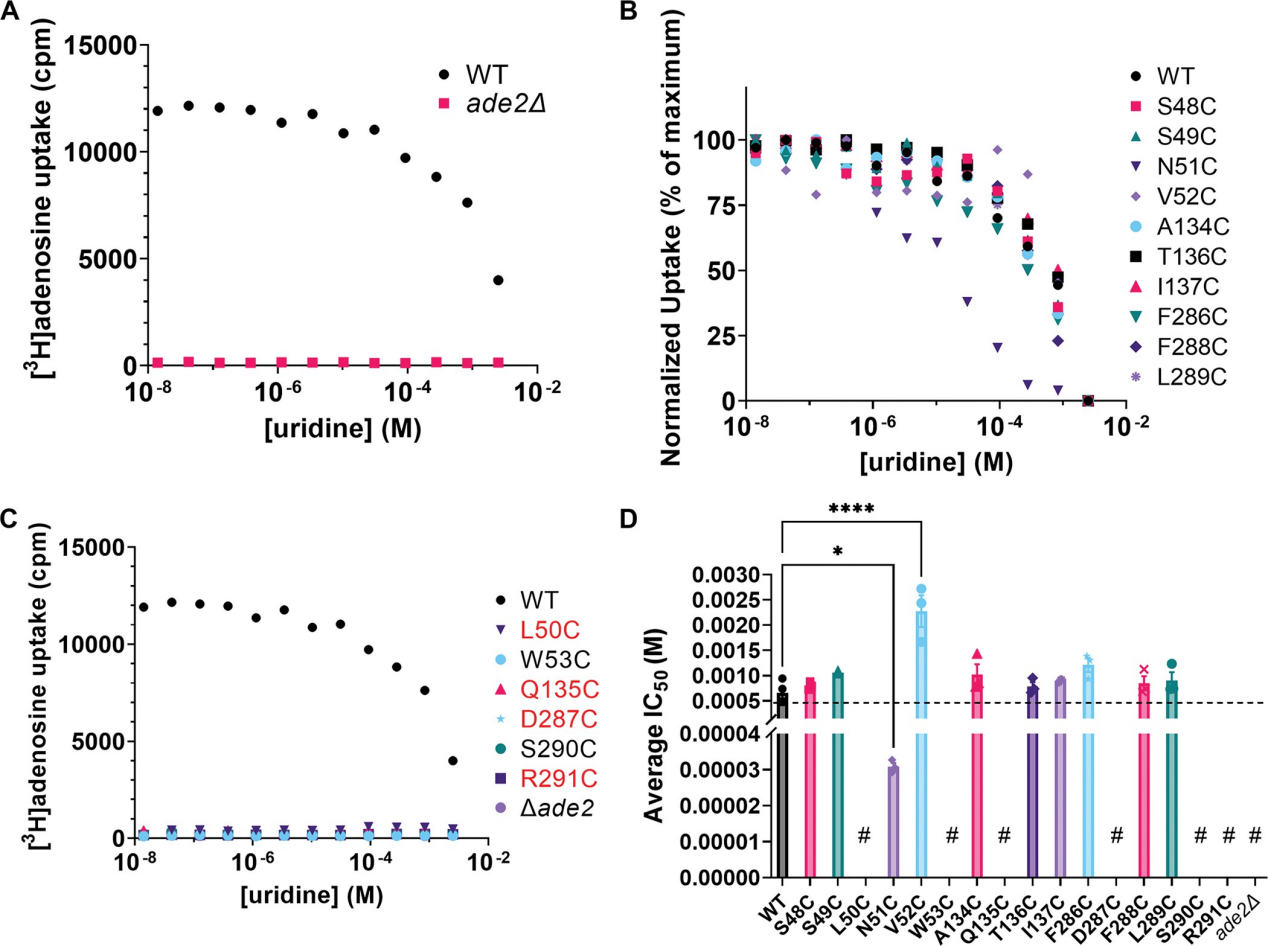
Assessment of WT and mutant PfENT1 function by [^3^H]adenosine uptake assay. (A) Representative experiments showing uridine competition with [^3^H]adenosine for the background purine auxotrophic yeast strain (*ade2*Δ*/fui1*Δ) (brown squares) and for the strain expressing WT PfENT1 (blue circles). The purine auxotrophic yeast (*ade2*Δ*/fui1*Δ*)* background strain did not import [^3^H]adenosine, while yeast expressing WT PfENT1 imported [^3^H]adenosine and displayed concentration-dependent inhibition of uptake by cold uridine. (B) Normalized [^3^H]adenosine uptake competition with cold uridine for all of the mutants that were functional in the adenosine-dependent proliferation assays, except L50C. These showed [^3^H]adenosine uptake and concentration-dependent inhibition of [^3^H]adenosine uptake by uridine. (C) Representative experiments showing lack of [^3^H]adenosine uptake for those PfENT1 mutants that were not functional in the adenosine-dependent proliferation assay and for L50C. Uptake by WT PfENT1 expressing yeast is shown as a positive control (blue circles). (D) Calculated IC_50_ values for uridine inhibition of [^3^H]adenosine uptake in the functional mutants and WT. Mean, SEM, and individual data points are shown (n≥3). Statistical significance was calculated by one-way ANOVA, (****, p<*0*.*0001*; *, *p = 0*.*027*). The # symbol indicates mutants that did not import [^3^H]adenosine.

The two yeast growth assays do not provide a direct measure of the effect of mutations on the affinity for transported substrates. To measure affinity for transported substrates we tested the ability of yeast expressing the various mutants to import tritiated adenosine or uridine in a 15-minute uptake assay that we have used previously [13, 14, 27]. Of note, in addition to transporting purines, PfENT1 can also transport pyrimidines, such as uridine [19, 28]. To ensure that competition between the tritiated substrate and the cold substrate only occurs at the transporter and not at a downstream metabolic enzyme, we used unlabeled adenosine to compete with [^3^H]uridine uptake (Fig 4) and unlabeled uridine to compete with [^3^H]adenosine uptake (Fig 5). Uridine and adenosine do not share common metabolic enzymes; thus, competition can only occur at the transporter [29, 30]. There was no uptake of either [^3^H]adenosine or [^3^H]uridine by the *ade2*Δ, *fui1*Δ purine auxotrophic background yeast. For yeast expressing WT PfENT1, the calculated IC_50_ values for adenosine and uridine were 101 μM and 997 μM, respectively (Figs 4 and 5, Table 1). For yeast expressing the mutants L50C, W53C, Q135C, D287C, S290C, and R291C, there was no uptake of either [^3^H]adenosine or [^3^H]uridine during the 15 min assay. We were unable to determine substrate IC_50_ values for these mutants. None of these mutants grew in the adenosine dependent growth assay, except L50C, which grew more slowly than WT and was killed in the 5-FUrd cytotoxicity assay (Figs 2 and 3, and Table 1). The adenosine concentration in the growth assays was 1 or 2 mM, much higher than the 50 nM concentration in the radioactive substrate uptake assay. In addition, the growth medium pH is about 4 during the 24 h assay, but the pH is 7.5 during the 15 min uptake assay. These differences in assay conditions may provide an explanation for the absence of uptake by the L50C mutant expressing yeast in the radioactive substrate uptake assays. For WT and the other mutants, Table 1 has the calculated IC_50_ values for the competition assay. All are similar to WT. Of note, yeast expressing the F286C mutant grew slowly in the adenosine-dependent growth assay (Fig 2) but had IC_50_ values similar to WT for both adenosine and uridine uptake (Figs 4 and 5, Table 1). Thus, the functional mutants did not significantly alter the affinity for adenosine. However, the uridine affinity was altered in the N51C and V52C mutants (Fig 5D). Neither of these residues interacts with the ribose sugar or the nucleobase in the PfENT1 cryo-EM structure [17]. It is possible that these mutations alter the local structure and the molecular interactions with the pyrimidine nucleoside.

### Comparison of current results with those of the cryo-EM structure of PfENT1

The structure of hENT1 and the AlphaFold generated PfENT1 structure are in outward open conformations (Fig 1) [15]. In contrast, the PfENT1 cryo-EM structure is in an inward open conformation. It was solved using a construct containing a Y190A mutation with nanobodies that bound in the cytoplasmic mouth of the transport pathway [17]. In the paper reporting the cryo-EM structure of PfENT1 they characterized the functional effects of mutation of five PfENT1 residues to alanine, S49, W53, A135, D287, and R291 [17]. Using isothermal calorimetry (ITC) they reported that inosine did not bind to the S49A, Q135A, and D287A mutants, and bound with lower affinity to the W53A and R291A mutants [17]. Despite ITC showing that inosine bound to W53A or R291A, yeast expressing either of these mutants or Q135A and D287A did not show uptake of radioactive inosine suggesting that none of these four mutants were functional in the Y190A background [17]. Consistent with these results, we found that W53C, Q135C, D287C, and R291C were not functional in either the yeast proliferation assay or the radioactive substrate uptake assays (Table 1). In contrast to the absence of inosine binding to the S49A mutant, we found that the S49C mutant was functional with similar affinity for adenosine and uridine to WT in the radioactive substrate uptake assays (Table 1). The amino acid found at position 49 is not conserved among ENT1 homologues. Both cysteine and threonine are found aligned at this position in S2 Fig. Thus, alanine may not be tolerated at this position but amino acids that can form hydrogen bonds are. We also found that the function of the L50C mutant was reduced. In the cryo-EM structure L50 is reported to limit the rotation of inosine but is not in direct contact [17].

## Conclusions

Our previous work has suggested that the malaria parasite purine uptake transporter, PfENT1, might be a good target for the development of novel antimalarial drugs and that our HTS assay effectively identifies PfENT1 inhibitors [13, 14]. In the current work, we show that the structural similarity between the AlphaFold predicted structure of PfENT1 and the crystal structure of hENT1 generates an amino acid sequence alignment that correctly aligns the purine nucleoside interacting residues in the two proteins. This was confirmed by high resolution PfENT1 structure that was recently published while this manuscript was in preparation [17]. Mutation of the residues that form hydrogen bonds to the purine nucleobase and ribose moieties, Q135, D287, and R291, resulted in loss of function of the resultant transporters. Consistent with the highly conserved PfENT1 residues D287 and R291 being important for substrate recognition and transport activity due to their interaction with the ribose OH groups, mutation of the aligned residues in the *Leishmania donovani* ENT2 (LdNT2) homologue also had a significant impact of transport function [31]. This supports the hypothesis that these residues perform a critical role in substrate recognition and transport.

Mutation of two additional surrounding residues to cysteine also resulted in a total loss of transport function, W53 and S290. It is notable that tryptophan is absolutely conserved at the position aligned with W53 in ENT1 homologues in a multiple species alignment of ENT1 homologues from a variety of species ranging from mammalian to protozoan (S2 Fig). The adjacent residue, N54 in PfENT1, is also absolutely conserved in the same multiple species sequence alignment. Mutation of a large aromatic tryptophan residue to cysteine might have significant local effects on protein structure that may alter the position of the nearby purine interacting residue, L50. Alternatively, the W53C mutation may alter the ability of the protein to undergo the conformational changes necessary for substrate translocation or may have altered protein folding leading to failure of the resultant protein to translocate to the plasma membrane. We cannot distinguish between these possibilities and believe that attempts to do so are beyond the scope of the current work. The total loss of function due to the S290C mutation was quite surprising because this is perhaps the most conservative amino acid substitution that one could make. S290 does lie in close proximity to the adjacent TM10 G355, which is in a very highly conserved region of this membrane-spanning segment. So it may play a critical structural role in the protein, though in hENT1 the residue aligned with S290 is a glycine. In contrast to the important role of residues in TM1, TM4, and TM8 in substrate recognition and protein function, mutation of multiple sequential residues to cysteine, one at a time, in TM2 (F63-V79) and TM10 (A348-F357) did not cause significant loss of PfENT1 function [32]. Cysteine-substitution mutants at the highly conserved TM11 glycine residues in the GXXXG motif found in all ENT1 homologues (PfENT1 mutants G392C and G396C) did result in an almost total loss of protein expression in a *Xenopus* oocyte expression system [21]. In the AlphaFold predicted PfENT1 structure, residues from TM7 interact with the TM11 helix backbone in the region of these two glycines. Thus, they must play an important role in maintaining the structure of the protein.

Despite the AlphaFold predicted structure being in outward-facing and PfENT1 cryoEM structure being in inward-facing conformations, the structures have similar overall structures in the transmembrane domains and a similar set of residues lining the substrate permeation pathway [17]. These structures will facilitate medicinal chemistry efforts to optimize PfENT1 inhibitors [14]. These inhibitors may serve as potential novel antimalarial drugs.

## Supporting information

S1 Fig 24 h growth curves for yeast expressing PfENT1 containing mutations to putative purine binding site residues with 10 mM adenosine in the growth media. For this assay, adenosine is the only purine source for the yeast (n = 5). (A) Growth of purine auxotrophic yeast (ade2Δ/fui1Δ) that do not (open squares) or do (closed squares) express WT PfENT1 in 10 mM adenosine. (B) Twenty-four-hour growth curves for yeast expressing various PfENT1 mutants in 10 mM adenosine. The yeast expressing PfENT1 containing mutations to putative purine binding site residues are highlighted with red arrows. The yeast expressing PfENT1 with W53C, Q135C, D287C, S290C, and R291C did proliferate slowly at 10 mM adenosine as did the purine auxotrophic background strain. We think that this proliferation is due to nucleosidase activity that converts adenosine to adenine which can then be imported via endogenous nucleobase transporters.
(TIF)

S2 Fig Multiple sequence alignment of 11 close homologues of hENT1 and 14 close homologues of PfENT1. Clustal Omega (https://www.ebi.ac.uk/Tools/msa/clustalo/) was used to align the sequences of hENT1, PfENT1 and 25 homologues (10 hENT1 similar sequences and 15 PfENT1 similar sequences) using the default alignment parameters. The sequence alignment of hENT1 and PfENT1 is consistent with the structure-based sequence alignment in Fig 1. The transmembrane segments from the x-ray crystal structure of hENT1 are highlighted in yellow. The residues mutated to cysteine in the present work are in orange. Residues mutated to cysteine in previous work are shown in red [32]. Protein sequences were retrieved from the National Library of Medicine server using the accession numbers shown in the left of each row.
(PDF)

## References

[1] Organization WH. World malaria report 2022: World Health Organization; 2022.

[2] Zekar L, Sharman T. Plasmodium Falciparum Malaria. StatPearls. Treasure Island (FL): StatPearls Publishing Copyright © 2023, StatPearls Publishing LLC.; 2023.

[3] Efficacy and safety of RTS,S/AS01 malaria vaccine with or without a booster dose in infants and children in Africa: final results of a phase 3, individually randomised, controlled trial. The Lancet. 2015;386(9988):31–45. doi: 10.1016/s0140-6736(15)60721-8 25913272 PMC5626001

[4] Olotu A, Fegan G, Wambua J, Nyangweso G, Leach A, Lievens M, et al. Seven-Year Efficacy of RTS,S/AS01 Malaria Vaccine among Young African Children. New England Journal of Medicine. 2016;374(26):2519–29. doi: 10.1056/NEJMoa1515257 27355532 PMC4962898

[5] Dondorp AM, Nosten F, Yi P, Das D, Phyo AP, Tarning J, et al. Artemisinin Resistance in *Plasmodium falciparum* Malaria. New England Journal of Medicine. 2009;361(5):455–67. doi: 10.1056/nejmoa0808859 19641202 PMC3495232

[6] Ashley EA, Dhorda M, Fairhurst RM, Amaratunga C, Lim P, Suon S, et al. Spread of Artemisinin Resistance in *Plasmodium falciparum* Malaria. New England Journal of Medicine. 2014;371(5):411–23. doi: 10.1056/nejmoa1314981 25075834 PMC4143591

[7] Balikagala B, Fukuda N, Ikeda M, Katuro OT, Tachibana S-I, Yamauchi M, et al. Evidence of Artemisinin-Resistant Malaria in Africa. New England Journal of Medicine. 2021;385(13):1163–71. doi: 10.1056/NEJMoa2101746 34551228

[8] Lu F, Culleton R, Zhang M, Ramaprasad A, Von Seidlein L, Zhou H, et al. Emergence of Indigenous Artemisinin-Resistant *Plasmodium falciparum* in Africa. New England Journal of Medicine. 2017;376(10):991–3. doi: 10.1056/nejmc1612765 28225668

[9] Cheviet T, Lefebvre-Tournier I, Wein S, Peyrottes S. *Plasmodium* Purine Metabolism and Its Inhibition by Nucleoside and Nucleotide Analogues. Journal of Medicinal Chemistry. 2019;62(18):8365–91. doi: 10.1021/acs.jmedchem.9b00182 30964283

[10] Bissati KE, Downie MJ, Kim S-K, Horowitz M, Carter N, Ullman B, et al. Genetic evidence for the essential role of PfNT1 in the transport and utilization of xanthine, guanine, guanosine and adenine by *Plasmodium falciparum*. Molecular and Biochemical Parasitology. 2008;161(2):130–9. doi: 10.1016/j.molbiopara.2008.06.012 18639591 PMC2612043

[11] El Bissati K, Zufferey R, Witola WH, Carter NS, Ullman B, Ben Mamoun C. The plasma membrane permease PfNT1 is essential for purine salvage in the human malaria parasite *Plasmodium falciparum*. Proceedings of the National Academy of Sciences. 2006;103(24):9286–91. doi: 10.1073/pnas.0602590103 16751273 PMC1482602

[12] Zhang M, Wang C, Otto TD, Oberstaller J, Liao X, Adapa SR, et al. Uncovering the essential genes of the human malaria parasite Plasmodium falciparum by saturation mutagenesis. Science. 2018;360(6388):eaap7847. doi: 10.1126/science.aap7847 29724925 PMC6360947

[13] Frame IJ, Deniskin R, Rinderspacher A, Katz F, Deng S-X, Moir RD, et al. Yeast-Based High-Throughput Screen Identifies *Plasmodium falciparum* Equilibrative Nucleoside Transporter 1 Inhibitors That Kill Malaria Parasites. ACS Chemical Biology. 2015;10(3):775–83. doi: 10.1021/cb500981y 25602169 PMC4369170

[14] Sosa Y, Deniskin R, Frame IJ, Steiginga MS, Bandyopadhyay D, Graybill TL, et al. Identification via a Parallel Hit Progression Strategy of Improved Small Molecule Inhibitors of the Malaria Purine Uptake Transporter that Inhibit *Plasmodium falciparum* Parasite Proliferation. ACS Infectious Diseases. 2019;5(10):1738–53. doi: 10.1021/acsinfecdis.9b00168 31373203 PMC7171677

[15] Wright NJ, Lee S-Y. Structures of human ENT1 in complex with adenosine reuptake inhibitors. Nature Structural & Molecular Biology. 2019;26(7):599–606. doi: 10.1038/s41594-019-0245-7 31235912 PMC6705415

[16] Jumper J, Evans R, Pritzel A, Green T, Figurnov M, Ronneberger O, et al. Highly accurate protein structure prediction with AlphaFold. Nature. 2021;596(7873):583–9. doi: 10.1038/s41586-021-03819-2 34265844 PMC8371605

[17] Wang C, Yu L, Zhang J, Zhou Y, Sun B, Xiao Q, et al. Structural basis of the substrate recognition and inhibition mechanism of *Plasmodium falciparum* nucleoside transporter PfENT1. Nature Communications. 2023;14(1). doi: 10.1038/s41467-023-37411-1 36977719 PMC10050424

[18] Pettersen EF, Goddard TD, Huang CC, Couch GS, Greenblatt DM, Meng EC, et al. UCSF Chimera?A visualization system for exploratory research and analysis. Journal of Computational Chemistry. 2004;25(13):1605–12. doi: 10.1002/jcc.20084 15264254

[19] Frame IJ, Deniskin R, Arora A, Akabas MH. Purine import into malaria parasites as a target for antimalarial drug development. Annals of the New York Academy of Sciences. 2015;1342(1):19–28. doi: 10.1111/nyas.12568 25424653 PMC4405406

[20] Murakami C, Kaeberlein M. Quantifying Yeast Chronological Life Span by Outgrowth of Aged Cells. Journal of Visualized Experiments. 2009;(27). doi: 10.3791/1156 19421136 PMC2762921

[21] Riegelhaupt PM, Frame IJ, Akabas MH. Transmembrane Segment 11 Appears to Line the Purine Permeation Pathway of the *Plasmodium falciparum* Equilibrative Nucleoside Transporter 1 (PfENT1). Journal of Biological Chemistry. 2010;285(22):17001–10. doi: 10.1074/jbc.m110.115758 20335165 PMC2878030

[22] Varadi M, Anyango S, Deshpande M, Nair S, Natassia C, Yordanova G, et al. AlphaFold Protein Structure Database: massively expanding the structural coverage of protein-sequence space with high-accuracy models. Nucleic Acids Research. 2022;50(D1):D439–D44. doi: 10.1093/nar/gkab1061 34791371 PMC8728224

[23] Akabas MH. Cysteine Modification: Probing Channel Structure, Function and Conformational Change. Adv Exp Med Biol. 2015;869:25–54. doi: 10.1007/978-1-4939-2845-3_3 26381939

[24] Winzeler EA, Shoemaker DD, Astromoff A, Liang H, Anderson K, Andre B, et al. Functional Characterization of the *S*. *cerevisiae* Genome by Gene Deletion and Parallel Analysis. Science. 1999;285(5429):901–6. doi: 10.1126/science.285.5429.901 10436161

[25] Wagner R, de Montigny J, de Wergifosse P, Souciet JL, Potier S. The ORF YBL042 of Saccharomyces cerevisiae encodes a uridine permease. FEMS Microbiology Letters. 1998;159(1):69–75. doi: 10.1111/j.1574-6968.1998.tb12843.x 9485596

[26] Frame IJ, Deniskin R, Rinderspacher A, Katz F, Deng S-X, Moir RD, et al. Yeast-Based High-Throughput Screen Identifies *Plasmodium falciparum* Equilibrative Nucleoside Transporter 1 Inhibitors That Kill Malaria Parasites. ACS Chemical Biology. 2015;10(3):775–83. doi: 10.1021/cb500981y 25602169 PMC4369170

[27] Sosa Y, Egbo D, Akabas MH. Impact of Field Isolate Identified Nonsynonymous Single Nucleotide Polymorphisms on *Plasmodium falciparum* Equilibrative Nucleoside Transporter 1 Inhibitor Efficacy. ACS Infectious Diseases. 2020;6(2):205–14. doi: 10.1021/acsinfecdis.9b00203 31876139 PMC9218960

[28] Parker MD, Hyde RJ, Yao Sym, Mcrobert L, Cass Ce, Young JD, et al. Identification of a nucleoside/nucleobase transporter from *Plasmodium falciparum*, a novel target for anti-malarial chemotherapy. Biochemical Journal. 2000;349(1):67–75. doi: 10.1042/bj3490067 10861212 PMC1221121

[29] Riegelhaupt PM, Cassera MB, Fröhlich RFG, Hazleton KZ, Hefter JJ, Schramm VL, et al. Transport of purines and purine salvage pathway inhibitors by the Plasmodium falciparum equilibrative nucleoside transporter PfENT1. Molecular and Biochemical Parasitology. 2010;169(1):40–9. doi: 10.1016/j.molbiopara.2009.10.001 19818813 PMC2783484

[30] Kirk K, Howitt SM, Bröer S, Saliba KJ, Downie MJ. Purine uptake in Plasmodium: transport versus metabolism. Trends in Parasitology. 2009;25(6):246–9. doi: 10.1016/j.pt.2009.03.006 19423394

[31] Arastu-Kapur S, Ford E, Ullman B, Carter NS. Functional Analysis of an Inosine-Guanosine Transporter from *Leishmania donovani*. Journal of Biological Chemistry. 2003;278(35):33327–33. doi: 10.1074/jbc.m305141200 12807872

[32] Nishtala SN, Arora A, Reyes J, Akabas MH. Accessibility of substituted cysteines in TM2 and TM10 transmembrane segments in the *Plasmodium falciparum* equilibrative nucleoside transporter PfENT1. Journal of Biological Chemistry. 2019;294(6):1924–35. doi: 10.1074/jbc.ra118.006547 30541922 PMC6369291

