# Supplementary figures and images for: Putative purine nucleoside interacting residues in the malaria parasite purine uptake transporter PfENT1 are critical for transporter function
Source: PLoS One. 2023 Dec 19;18(12):e0293923. doi: 10.1371/journal.pone.0293923 (PMC10729961; doi:10.1371/journal.pone.0293923)

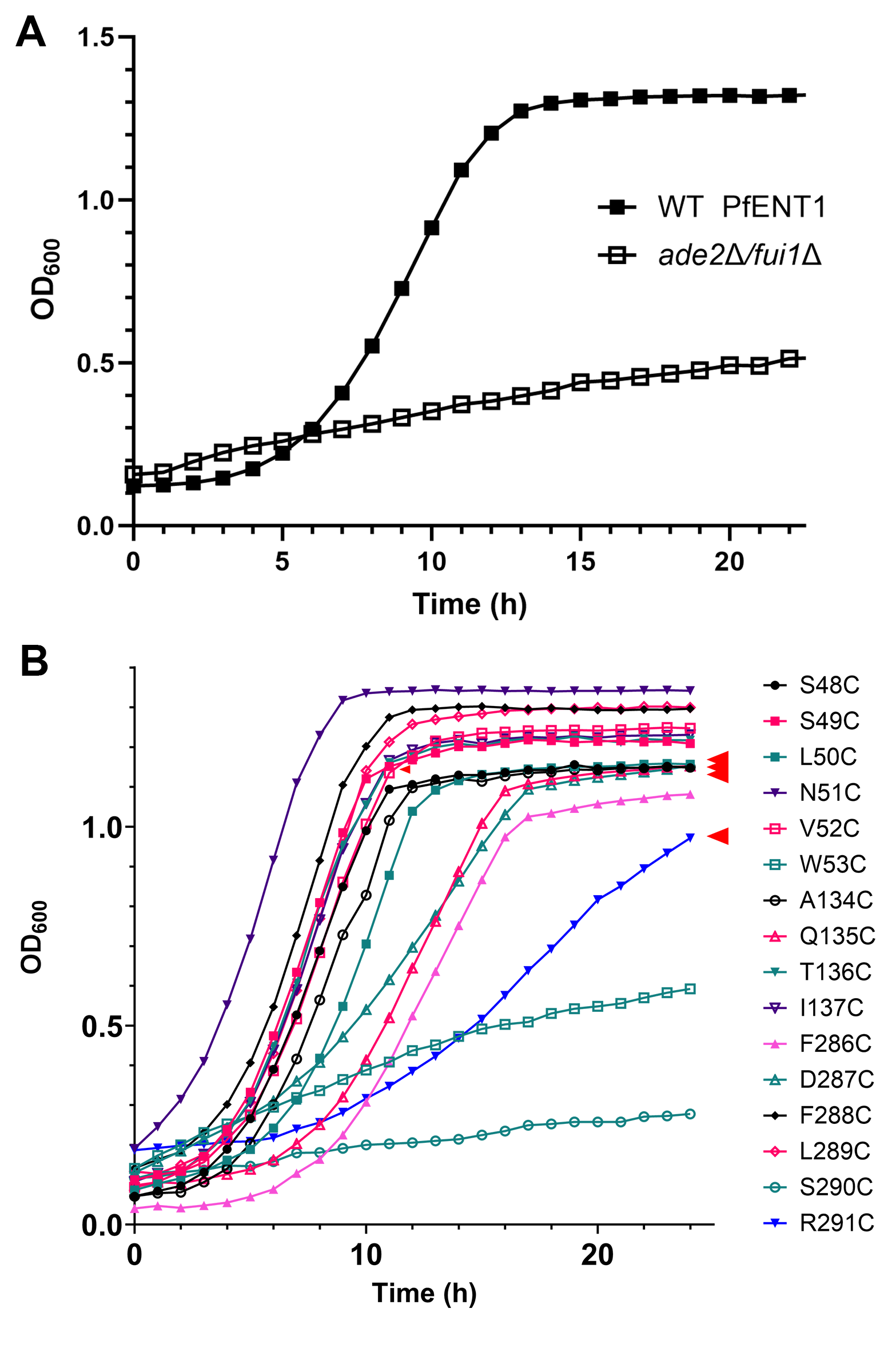

Supplement: S1 Fig — For this assay, adenosine is the only purine source for the yeast (n = 5). (A) Growth of purine auxotrophic yeast (ade2Δ/fui1Δ) that do not (open squares) or do (closed squares) express WT PfENT1 in 10 mM adenosine. (B) Twenty-four-hour growth curves for yeast expressing various PfENT1 mutants in 10 mM adenosine. The yeast expressing PfENT1 containing mutations to putative purine binding site residues are highlighted with red arrows. The yeast expressing PfENT1 with W53C, Q135C, D287C, S290C, and R291C did proliferate slowly at 10 mM adenosine as did the purine auxotrophic background strain. We think that this proliferation is due to nucleosidase activity that converts adenosine to adenine which can then be imported via endogenous nucleobase transporters. (TIF) [file pone.0293923.s001.tif]
