# Supplementary material for: Putative purine nucleoside interacting residues in the malaria parasite purine uptake transporter PfENT1 are critical for transporter function
Source: PLoS One. 2023 Dec 19;18(12):e0293923. doi: 10.1371/journal.pone.0293923 (PMC10729961; doi:10.1371/journal.pone.0293923)
Supplement: S2 Fig — Clustal Omega (https://www.ebi.ac.uk/Tools/msa/clustalo/) was used to align the sequences of hENT1, PfENT1 and 25 homologues (10 hENT1 similar sequences and 15 PfENT1 similar sequences) using the default alignment parameters. The sequence alignment of hENT1 and PfENT1 is consistent with the structure-based sequence alignment in Fig 1. The transmembrane segments from the x-ray crystal structure of hENT1 are highlighted in yellow. The residues mutated to cysteine in the present work are in orange. Residues mutated to cysteine in previous work are shown in red [32]. Protein sequences were retrieved from the National Library of Medicine server using the accession numbers shown in the left of each row. (PDF) [file pone.0293923.s002.pdf]

```
XP_004935447.1      --------------------------MTTRDGPQDRYKAVWLIFFILGLGTLLPWNFFMT    34
NP_001025348.1      --------------------------MTAVNAPRDRYNAVWIIFFILGLGTLLPWNFFMT    34
NP_113872.1         --------------------------MTTSHQPQDRYKAVWLIFFVLGLGTLLPWNFFIT    34
AAH04828.1          --------------------------MTTSHQPQDRYKAVWLIFFVLGLGTLLPWNFFMT    34
XP_003128473.4      -------MQAPEGGSSQPGITENAVTMTTSHQPQDRYRAVWLIFFMLGLGTLLPWNFFMT    53
XP_005223466.1      --------------------------MTTSHQPQDRYKAVWLIFFILGLGTLLPWNFFMT    34
XP_010861397.1      --------------------------MTTSHQPQDRYKAVWLIFFILGLGTLLPWNFFMT    34
XP_006931832.1      --------------------------MTTGHEPQDRYKAVWLIFFMLGLGTLLPWNFFMT    34
XP_016068296.1      --------------------------MTTSHQPQDRYKAVWLIFFMLGLGTLLPWNFFMT    34
XP_017402910.1      --------------------------MTTSHQPQDRYKGVWLIFFMLGLGTLLPWNFFMT    34
hENT1               --------------------------MTTSHQPQDRYKAVWLIFFMLGLGTLLPWNFFMT    34
XP_003311378.1      MLTPKSQQQAPEGGSCQPGKTENTITMTTSHQPQDRYKAVWLIFFMLGLGTLLPWNFFMT    60
XP_022813752.1      MSKIKESSSGILGS--------N-NTNKESSKKNAGSIALPITYALIGVSCLNVWNSALG    51
XP_022713821.1      MSKIKESSSGILGA-------SN-NTNKESSQKSARSIALPMTYALIGVSCLNVWNSALG    52
EUD71780.1          MSEIKESSSGILGS-------SN-NTKKGSPQRPSTSMALPITYALIGVSCLNVWNTALG    52
SCM11350.1          MSEIKESSNGFLGS-------SN-NANKGSSQRPGVSIALPITYILIGVSCLNVWNSALG    52
SCM09553.1          MSEIKESSNGFLGS-------SN-NANKGSSQRPTTSIALPLTYILIGVSCLNVWNSALG    52
XP_028534619.1      MSVNRDLSKVSSDIEMKEVYMDD-HRKELKATNKKKHYLNILTFILIGLSSLNVWNTALG    59
XP_028527991.1      MSTGKDSSKVSADIEMKEVYVDE-NKKSSKITSTNKRTLNILTFILIGLSSLNVWNTALG    59
PfENT1-3D7          MSTGKESSKAYADIESRGD-YKD-DGKKGSTLSSKQHFMLSLTFILIGLSSLNVWNTALG    58
XP_028862871.1      MSTSKQASKALVDIEKKGGDSKC-DDKQGSKLNKREQIILPLTFILIGLSSLNVWNTALG    59
SBT77967.1          MSTGKESSKTFVDIEKK-GGDYK-DGKGWSGLSKRQEYILPFTFILIGLSSLNVWNTALG    58
SBS85808.1          MSTGKESSKTFVDIEKKGGGDYK-DGKGWSGLSKRQQYILPFTFILIGLSSLNVWNTALG    59
XP_028544692.1      MSISKESSKTMLDIEKKAG-EKK-DFKDVSKLNKKEQLILPFTFLLIGLSSLNVWNTALG    58
XP_002259877.1      MSISKESSNTMIDIERKAG-EGK-EGEDVSKWSKNERFLFPLTFILIGLGSLNVWNTALG    58
XP_019916700.1      MSISKESSNTMIDIERKAG-EGK-DGDSGSKLTKNEKFLFPLTFILIGLSSLNVWNTALG    58
XP_001614251.1      MSISKESSKTMIDIEKKGG-EGK-DGKGGSKMTKNEQFLLPFTFILIGLSSLNVWNTALG    58
                                                             : : ::*:. *  **  :

XP_004935447.1      ARQYFINRLADPQNISHLSNQTS----------------VGTASDLSYLQSMFDNFMTLC    78
NP_001025348.1      ATLYFTKRLEETNGGLN--------------------QTANTTEIRSVLQSKFNNVMTLC    74
NP_113872.1         ATQYFTSRLNTSQNISLVTNQS-------CESTEALADPSVSLPARSSLSAIFNNVMTLC    87
AAH04828.1          ATKYFTNRLDVSQNVSSDTDQS-------CESTKALADPTVALPARSSLSAIFNNVMTLC    87
XP_003128473.4      ATAYFTNRLDMSHNVSLGPAELSKDVELLATSTAPLATSTTPSTERNYLSAIFNNVMTLC    113
XP_005223466.1      ATKYFTNRLDMSQNMSLGPAEVSKDIQASA-------SPLAPSPERTHLSTIFNNVMTLC    87
XP_010861397.1      ATKYFTNRLDMSQNMSLGPAEVSKDIQASA-------SPLAPSPERTHLSTIFNNVMTLC    87
XP_006931832.1      ATRYFTNRLDETQNMSLVTAENSKDFQPSA-------TPTVPSPERNYLSALFNNVMTLC    87
XP_016068296.1      ATQYFTNRLDQSQNVSLVTAELSRGIQASV-------TPTTPSAERNSLSAIFNNVMTLC    87
XP_017402910.1      ATQYFTSRLDMPQNVSLVTAELSKDAQASA-------APTAPLTERNSLSAIFNNVMTLC    87
hENT1               ATQYFTNRLDMSQNVSLVTAELSKDAQASA-------APAAPLPERNSLSAIFNNVMTLC    87
XP_003311378.1      ATQYFTNRLDMSQNVSLVTAELSKDAQASA-------APAAPLPERNSLSAIFNNVMTLC    113
XP_022813752.1      LNIKIKYNIFQMAG-----------------------------LLTSSVLALFVNYPR--    80
XP_022713821.1      LNIKITYNIFQMAG-----------------------------LLTSSVLALFVNYPR--    81
EUD71780.1          LNIKITYNIFQMAG-----------------------------LLGSSVLSLFVNYPR--    81
SCM11350.1          LNIKIKYNIFQMAG-----------------------------LLSSAVISIFVNYPR--    81
SCM09553.1          LNIKITYNIFQMAG-----------------------------LLSSAVLSIFVNYPR--    81
XP_028534619.1      LNINLKYNAFQMTG-----------------------------LVCSSIVALFIDIPK--    88
XP_028527991.1      LNINLKYNTFQITG-----------------------------LVCSSIVALFIDIPK--    88
PfENT1-3D7          LNINFKYNTFQITG-----------------------------LVCSSIVALFVEIPK--    87
XP_028862871.1      LNINFKYNTYQITG-----------------------------LVCSSIIALFINIPK--    88
SBT77967.1          LNINFKYNTFQITG-----------------------------LVCSSIIALFINIPK--    87
SBS85808.1          LNINFKYNTFQITG-----------------------------LVCSSIIALFINIPK--    88
XP_028544692.1      LNINFKYNTFQITG-----------------------------LVVSSIIALFVNLPK--    87
XP_002259877.1      LNINFKYNTFQITG-----------------------------LVCSSIIALFIKVPK--    87
XP_019916700.1      LNINFKYNTFQITG-----------------------------LVCSSIIALFIKVPK--    87
XP_001614251.1      LNINFKYNTFQITG-----------------------------LVCSSIIALFVKVPK--    87
                        :  .     .                                . : : * .
```

```
XP_004935447.1          SMVPLLIFTCLNSFIHQRIPQQIRISGSLVAIGLVFLITAIMVKVTMDPLPFFVFTMVSI    138
NP_001025348.1          AMVPLLIFTCLNSFIHQRIPQKLRISGSLSVILVVFLITAVLVKVEMEPLPFFTLTMIKI    134
NP_113872.1             AMLPLLIFTCLNSFLHQKVSQSLRILGSLLAILLVFLVTATLVKVQMDALSFFIITMIKI    147
AAH04828.1              AMLPLLVFTCLNSFLHQRISQSVRILGSLLAILLVFLVTAALVKVEMDALTFFVITMIKI    147
XP_003128473.4          AMLPLLLFTCLNSFLHQRIPQSVRILGSLVAILLVFLITAILVKVSLDPLPFFVITMIKI    173
XP_005223466.1          AMVPLLIFTCLNSFLHQRIPQSVRILGGLVAILLVFLITAILVKVPLHALSFFVITMLKI    147
XP_010861397.1          AMVPLLIFTCLNSFLHQRIPQSVRILGGLVAILLVFLITAILVKVPLHALSFFVITMLKI    147
XP_006931832.1          AMLPLLFFTCLNSFLHQRIPQSVRILGSLIAILLVFLITAVLVKVHLDAVSFFVITMIKI    147
XP_016068296.1          AMLPLLLFTYLNSFLHQRIPQSVRILGSLVAILLVFLITAVLVKVHLDALPFFIITMIKI    147
XP_017402910.1          AMLPLLLFTCLNSFLHQRIPQSVRILGSLVAILLVFLITAILVKVQLDPLPFFVITMIKI    147
hENT1                   AMLPLLLFTYLNSFLHQRIPQSVRILGSLVAILLVFLITAILVKVQLDALPFFVITMIKI    147
XP_003311378.1          AMLPLLLFTYLNSFLHQRIPQSVRILGSLVAILLVFLITAILVKVQLDALPFFVITMIKI    173
XP_022813752.1          -----VL-----------LP---SSLGVLTLLCAGFQIAHQT----FSDSAFDTYCLAAF    117
XP_022713821.1          -----VL-----------LP---TSLGVLTLLCAGFQIAHQT----FSDSAFDTYCLAAF    118
EUD71780.1              -----AL-----------LP---TSLGVLSLLCAGFQIAHQT----FSENAFDAYCLAAF    118
SCM11350.1              -----VM-----------LP---TSLGVLSLLCAGFQIAHQT----FSEAAFDAYCLAAF    118
SCM09553.1              -----FM-----------LP---TSLGILSLLCAGFQIAHQT----FSETAFDAYCLAAF    118
XP_028534619.1          -----KF-----------LP---YFLGALAILSAGFQISHQF----LSESQFSIYCLITF    125
XP_028527991.1          -----FL-----------LP---YFLGGLAILSAGFQITHQL----LTENQFSIYCLITF    125
PfENT1-3D7              -----IM-----------LP---FLLGGLSILCAGFQISHSF----FTDTQFDTYCLVAF    124
XP_028862871.1          -----IL-----------LP---YILGMLAILCGGFQISHRF----FEYKEFDTYCLVAF    125
SBT77967.1              -----LL-----------LP---YCLGGLAMLCGGFQIAHRY----FSFYYFDKYCLIAF    124
SBS85808.1              -----LL-----------LP---YCLGGLAMLCGGFQIAHRC----LSYYYFDKYCLIAF    125
XP_028544692.1          -----IL-----------LP---IALGGLAILCAGFQIAHQY----FTFEQFDTYCLIAF    124
XP_002259877.1          -----IL-----------LP---FTLGGLAALCAGFQVAHQF----FTAEQFDTYCLMAF    124
XP_019916700.1          -----ML-----------LP---FALGGLAILCAGFQIAHQF----FTFEQFDTYCLIAF    124
XP_001614251.1          -----ML-----------LP---FALGGLAMLCAGFQIAHQC----FTFEQFDTYCLIAF    124
                              .           :       * *  :   * ::       :    *    :  :

XP_004935447.1          VFINSFGAMLQGSLFGLAGLLPASYTAPIMSGQGLAGIFAALAMII-----SISIGAQQP    193
NP_001025348.1          ICINSFGAILQGSLFGLAGMLPASYTTPIMSGQGLAGAFAAFSMIC-----AIASGSELE    189
NP_113872.1             VLINSFGAILQASLFGLAGVLPANYTAPIMSGQGLAGFFTSVAMIC-----AVASGSKLS    202
AAH04828.1              VLINSFGAILQASLFGLAGVLPANYTAPIMSGQGLAGFFTSVAMIC-----AIASGSELS    202
XP_003128473.4          MLINSFGAILQGSLFGLAGLLPASYTAPIMSGQGLAGFFASVAMIC-----AIASGSELS    228
XP_005223466.1          MLINSFGAILQGSLFGLAGLLPASYTAPIMSGQGLAGFFASVAMIC-----AIASGSELS    202
XP_010861397.1          MLINSFGAILQGSLFGLAGLLPASYTAPIMSGQGLAGFFASVAMIC-----AIASGSELS    202
XP_006931832.1          MLINSFGAILQGSLFGLAGLLPTSYTAPIMSGQGLAGFFASVAMIC-----AIASGSELS    202
XP_016068296.1          MLINSFGAILQGSLFGLAGLLPASYTAPIMSGQGLAGIFASVAMIC-----AIASGSELS    202
XP_017402910.1          MLINSFGAILQGSLFGLAGLLPASYTAPIMSGQGLAGFFASVAMIC-----AIASGSELS    202
hENT1                   VLINSFGAILQGSLFGLAGLLPASYTAPIMSGQGLAGFFASVAMIC-----AIASGSELS    202
XP_003311378.1          VLINSFGAILQGSLFGLAGLLPASYTAPIMSGQGLAGFFASVAMIC-----AIASGSELS    228
XP_022813752.1          ITIGLMAGIAQTIAFAIGTTKESNMSGYISAGIGMSGVLIFCINLALDYIVSEAKKYGIN    177
XP_022713821.1          ITIGLMAGIAQTIAFAIGTTKESNMSGYISAGIGMSGVLIFCINLILDYIVSDEKIYEIN    178
EUD71780.1              ITIGLMAGIAQSIAFAIGTTKESNMSGYLSAGIGMSGVLIFCINLMLDYIVSAEKKYEIN    178
SCM11350.1              ITIGLMAGIAQSIAFAIGTTKENNMSGYLSAGVGMSGVLIFCINLILDCIVSTEKQFEIN    178
SCM09553.1              ITIGLMAGIAQSIAFAIGTTKENNMSGYLSAGVGMSGVLIFGINLILDCIVSTEKQFEIN    178
XP_028534619.1          IIIGIMAGLAQTIAFNIGSTMKENMGGYMSAGIGISGVFIFVINLLLDQFVSTEKKYGIN    185
XP_028527991.1          VIIGIMAGLAQTIAFNIASTMEENMGGYMSTGIGISGVFIFVINLLLDQFVSSEKKYGIN    185
PfENT1-3D7              IVIGVVAGLAQTIAFNIGSTMEDNMGGYMSAGIGISGVFIFVINLLLDQFVSPEKHYGVN    184
XP_028862871.1          IVIGIMAGFAQTIAFSIGTTMEENMGGYMSAGIGISGVFIFVINILLDLIVSDTKIHDIN    185
SBT77967.1              IVIGIMAGLAQTIAFSIGTTMENNMGGYMSAGIGISGVFIFVINLLLDQVVPDKKLYNVN    184
SBS85808.1              IVIGIMAGLAQTIAFSIGTTMENNMGGYMSAGIGISGVFIFVINLLLDQVVPDKKLYNVN    185
XP_028544692.1          ITIGILAGFVQTIAFNIGTTMEKNMGGYMSTGIGVSGVFIFIINLLLDQIVSEEKLYNVN    184
XP_002259877.1          IIIGIMAGLTQTIAFSVGTTMKENMGGYISAGFGISGVFIFIINLLLDQIVPNTKKYGVN    184
XP_019916700.1          IIIGIMAGLAQTIAFSVGTTMKENMGGYMSAGIGISGVFIFIINLILDQIVPETKKYGVN    184
XP_001614251.1          IVIGIMAGLAQTIAFSVGTTMEENMGGYMSAGIGISGVFIFIINLLLDQIVPDQKKFNVN    184
                        : *. ...: *   * :.     .    : :* *::* :     :
```

```
XP_004935447.1      ESYIGYFTTACVA-ILLAIFSYVLLPRMDFFRYYSMKDKTEYHVCNAELETKRDLIKKDE    252
NP_001025348.1      DSAFGYFITACVV-ILLAIVSYLALPRMEFFQYYSESNRSR---SSTDEENKMDLLKP--    243
NP_113872.1         ESAFGYFITACAV-VILAILCYLALPWMEFYRHYLQLNLAG---P-AEQETKLDLISE--    255
AAH04828.1          ESAFGYFITACAV-VILAILCYLALPRTEFYRHYLQLNLAG---P-AEQETKLDLISK--    255
XP_003128473.4      ESAFGYFITACAV-IILTIICYLGLPRLEFYRYYQQLKLEG---P-GEQETKLDLITK--    281
XP_005223466.1      ESAFGYFITACGV-IILTIICYLGLPRLEFYRYYRQLKLEG---P-GEQETKLDLISK--    255
XP_010861397.1      ESAFGYFITACGV-IILTIICYLGLPRLEFYRYYRQLKLEG---P-GEQETKLDLISK--    255
XP_006931832.1      ESAFGYFITACGV-IVLAIICYLGLPRLEFYRYYQQLKLEG---P-GEQETKLDLISK--    255
XP_016068296.1      ESAFGYFITACGV-IIVTIICYLGLPRLEFYRYYQQLKLEG---P-GEQETKLDLISK--    255
XP_017402910.1      ESAFGYFITACAV-IILNIICYLGLPRLEFYRYYQQLKLEG---P-GEQETKLDLISKES    257
hENT1               ESAFGYFITACAV-IILTIICYLGLPRLEFYRYYQQLKLEG---P-GEQETKLDLISK--    255
XP_003311378.1      ESAFGYFITACAV-IILTIICYLGLPRLEFYRYYQQLKLEG---P-GEQETKLDLISK--    281
XP_022813752.1      KSKLLILFSVSEIFLIVTIVCCVL---------------------------YIDLFPKND    210
XP_022713821.1      KSKLLCLFSISEIFLIITIVCCVL---------------------------YIDLFPKND    211
EUD71780.1          KAKLLWLFGISEVFLVVTIICCLV---------------------------YIDIFPKNS    211
SCM11350.1          KAKLLWLFGISEVFLVISVVCCLM---------------------------YIDIFPKND    211
SCM09553.1          KAKLLWLFGISEVFLVITIICCLM---------------------------YIDIFPKND    211
XP_028534619.1      REKLLYLYFICEICLILAIILCVF---------------------------NLELTKIKV    218
XP_028527991.1      KEKLLYLYFICEICLVLAILLCVF---------------------------NLELSKKKV    218
PfENT1-3D7          KAKLLYLYIICELCLILAIVFCVC---------------------------NLDLTNKNN    217
XP_028862871.1      AAKLLYLYIICEVCLVLAIIFCVC---------------------------NLELSSTST    218
SBT77967.1          EAKLLYLFIICELCLVLAIFFSVF---------------------------NLELSSNND    217
SBS85808.1          EAKLLYLFIICELCLVLAIFFSVF---------------------------NLELSSNKD    218
XP_028544692.1      KAKLLYLYFICEICLILSIIFSLC---------------------------NLELTSSKL    217
XP_002259877.1      EAKLLYLFIICEVCLLLAIIFSVC---------------------------NLELSSSKA    217
XP_019916700.1      KAKLLYLFIICELCLVLAIIFSVC---------------------------NLELSSSKE    217
XP_001614251.1      EAKLLYLFLICELCLVLAIIFSVC---------------------------NLELSSSKT    217
                       :  :   .   ::: :.  :                              ::

XP_004935447.1      ----------PNGMEQNNSKIIP-VHNPDEKPSVISIFKKLWVMAVSVCLVFTVTIGVFP    301
NP_001025348.1      -----------EGQAEKRPVLSLTEEESKPTVSVFAIFKQIWVMALSVCFVFIITIGIFP    292
NP_113872.1         -------GEEPRGGREESGVPGPNSLPANRNQSIKAILKSIWVLALSVCFIFTVTIGLFP    308
AAH04828.1          -------GEEPKGRREESGVPGPNSPPTNRNQSIKAILKSICVPALSVCFIFTVTIGLFP    308
XP_003128473.4      -------GEESKAGQEELRVSASNSQPSNKSHSVRAILRSILVPALSVCFVFTVTIGVFP    334
XP_005223466.1      -------GEESKAGQEETGFSAPSSQPAKESHSVRAILKSILVPAFSVCFVFTITIGIFP    308
XP_010861397.1      -------GEESKAGQEETRFSAPSSQPAKESHSVRAILKSILVPAFSVCFVFTITIGIFP    308
XP_006931832.1      -------GEDLKANKEESRVPAPNSESTNQGHSIRAILRNILVPALSVCFIFTVTIGMFP    308
XP_016068296.1      -------GEEPKAGKAESGVSAPNSLPTNKSHSIRAILRNILVPALSVCFIFTVTIGVFP    308
XP_017402910.1      STTCHPAGEEPRAGKEESGVSVSHSQATNESHSIKAILKNISVLAFSVCFIFTITIGMFP    317
hENT1               -------GEEPRAGKEESGVSVSNSQPTNESHSIKAILKNISVLAFSVCFIFTITIGMFP    308
XP_003311378.1      -------GEEPRAGKEESGVSVSNSQPTSESHSIKAILKNISVLAFSVCFIFTITIGMFP    334
XP_022813752.1      NNKD--------------STDIEKAEEKEGRLSLIEILKDGYKAILSIFLVNWLSLQLFP    256
XP_022713821.1      NNKD--------------STDIEKAEEKEGRLPLIEIIKDGYKAILSIFLVNWLSLQLFP    257
EUD71780.1          NN-D--------------SADIEKAEEKEDRLPLIEILKDGYKAILAIFLVNWLSLQLFP    256
SCM11350.1          NN-D--------------STDVEKAEEKEERLPFIEILKDGYKAILAIFLVNWLSLQLFP    256
SCM09553.1          NN-D--------------STDVEKGEEKEERLPFIEILKDGYKAILAIFLVNWLSLQLFP    256
XP_028534619.1      KK-------------------DDDS-ISERSLSYMELFKDSYKAIITIFFVNWLTLQLFP    258
XP_028527991.1      KK-------------------DDEE-ISERSLSYMELIKDSYKAIISIFFVNMLTLQLFP    258
PfENT1-3D7          KK-------------------DEENKENNATLSYMELFKDSYKAILTMFLVNWLTLQLFP    258
XP_028862871.1      KK-------------------EEEK-NDEPGLSYMELIKDSYKAILAMFLVNWLSLQLFP    258
SBT77967.1          KK-------------------EEEDSSKEEGLSYMELIKDSYKAILAMFLVNWLSLQLFP    258
SBS85808.1          KK-------------------EEEQNSKEEGLSYMELIKDSYKAILAMFLVNWLSLQLFP    259
XP_028544692.1      LK-------------------EEECNDKEPGLSYVELIKDSYKAILSMFLVNWLSLQLFP    258
XP_002259877.1      SK-------------------EEEYNDKEAGLSYWELIKDSYKAILAMFLVNWLSLQLFP    258
XP_019916700.1      SK-------------------EEEYNDKEQGLSYWELIKDSYKAILAMFLVNWLSLQLFP    258
XP_001614251.1      SK-------------------EEEYSDKEQGLSYLELLKDSYKAILAMFLVNWLSLQLFP    258
                                            .       :::.     .:: ::  ::: :**
```

```
XP_004935447.1      SITAKVSTTLGK-ESKWDLYFVSVSCFLIFNVFDWMGRSLTAL-----FTWPGKDSCLLP    355
NP_001025348.1      AVTVEVQSTIPD-RGAWEKYFIPVSCFLLFNVMDWVGRSLTAV-----CMWPGKDSIWLP    346
NP_113872.1         AVTAEVESSIAGTSPWKNCYFIPVACFLNFNVFDWLGRSLTAI-----CMWPGQDSRWLP    363
AAH04828.1          AVTAEVESSIAGTSPWKS-YFIPVACFLNFNVFDWLGRSLTAV-----CMWPGQDSRWLP    362
XP_003128473.4      AVAAEVKSSIADTTSPWNNYFIPVSCFLTFNIFDWLGRSLTAV-----TMWPGKDSLWLP    389
XP_005223466.1      AVTAEVESTIAG-TSAWKAYFIPVSCFLTFNVFDWLGRSLTAI-----TMWPGKDSYWLP    362
XP_010861397.1      AVTAEVESTIAG-TSAWKAYFIPVSCFLTFNVFDWLGRSLTAI-----TMWPGKDSYWLP    362
XP_006931832.1      AVTAEVQSSIAG-NSAWGAYFIPVSCFLTFNVFDWLGRSLTAI-----FTWPGKDSHWLP    362
XP_016068296.1      AITAEVKSSIGG-SSAWGHYFIPVSCFLTFNVFDWLGRSLTAI-----SMWPGKDSRWLP    362
XP_017402910.1      AVAVEVKSSIAG-TSAWEHYFIPVSCFLTFNIFDWLGRSLTAV-----FMWPGKDSRWLP    371
hENT1               AVTVEVKSSIAG-SSTWERYFIPVSCFLTFNIFDWLGRSLTAV-----FMWPGKDSRWLP    362
XP_003311378.1      AVTVEVKSSIAG-SSTWERYFIPVSCFLTFNIFDWLGRSLTAV-----FMWPGKDSRWLP    388
XP_022813752.1      GIGHKKWQESHGMT----DNNVTI-IVGMFQVFDFISRYPPNFTHIKIFKYFTFSLNTLL    311
XP_022713821.1      GIGHKKWQEKHGMT----DNNVTI-IVGMFQVFDFISRYPPNFTHIKIFKYFTFSLNTLL    312
EUD71780.1          GIGHKKWQESHGMS----DNHVTI-IVGMFQVFDFISRYPPNLTHIKMFKYFTFSLNTLL    311
SCM11350.1          GIGHKKWQQSHGMT----DNNVTI-IVGMFQVFDFVSRYPPIWSHMAIFKYFTFSLNTLL    311
SCM09553.1          GIGHKKWQESHGMP----DTHVTI-IVGMFQLFDFVSRYPPIWGHIPIFKYFTFSLNTLL    311
XP_028534619.1      GVGHKKWQESHDIS----DNNVTI-IVGMFQVFDFISRYPPNLSHMKYMKFFTFSLNKLL    313
XP_028527991.1      GVGHKKWAKSHDIS----DYNVTI-IVGMFQVFDFISRYPPNLSHIKYFKIFTFSLNKLL    313
PfENT1-3D7          GVGHKKWQESHNIS----DYNVTI-IVGMFQVFDFLSRYPPNLTHIKIFKNFTFSLNKLL    313
XP_028862871.1      GVGHKKWQQRHKIT----DYYVTI-IVGMFQVFDFVSRYPPNLSHIKIFKFFTFSLNKLL    313
SBT77967.1          GVGHKKWQESHGIS----DYYVTV-IVGMFQVFDFISRYPPNLSHIKYFKFFTFSLNKLL    313
SBS85808.1          GVGHKKWQESHGIS----DYYVTV-IVGMFQVFDFISRYPPNLSHIKFFKFFTFSLNKLL    314
XP_028544692.1      GVGHKKWQQSHGIS----DYNVTV-IVGMFQVFDFVSRYPPNLSHIKFFKFFTFSLNKLL    313
XP_002259877.1      GVGHKKWQQSHNIS----DNKVTL-IVGMFQVFDFISRYPPNLSHMKIFKCFTFSLNKLL    313
XP_019916700.1      GVGHKKWQQSHNIS----DYNVTL-IVGMFQVFDFVSRYPPNLSHMKIFKCFSFSLNKLL    313
XP_001614251.1      GVGHKKWQESHNIS----DYNVTL-IVGMFQVFDFVSRYPPNLSHMKIFKWFTFSLNKLL    313
                    .:  :                : :  .  *:::*::.*                .   *

XP_004935447.1      VMVVLRVIFIPLFMLCNVQPRNHL----PVIFSHDAWYIIFMIFFSISNGYLASLCMCFG    411
NP_001025348.1      ILVIARVVFVPLFILCNVQPRSFL----PVVFSHDAWYIIFMIFFSFSNGYLASLCMCFG    402
NP_113872.1         VLVACRVVFIPLLMLCNVKQHHYL----PSLFKHDVWFITFMAAFAFSNGYLASLCMCFG    419
AAH04828.1          VLVASRIVFVPLLMLCNVKARHCGAQRHHFVFKHDAWFIAFMAAFAFSNGYLASLCMCFG    422
XP_003128473.4      ILVLARLAFVPLLLLCNVQPRHYL----PMVFDHDALYIFFMAAFAFSNGYLASLCMCFG    445
XP_005223466.1      SLVLARLAFVPLLLLCNVQPRRNL----PVVFEHDSWFIIFMAAFAFSNGYLASLCMCFG    418
XP_010861397.1      SLVLARLAFVPLLLLCNVQPRRNL----PVVFEHDSWFIIFMAAFAFSNGYLASLCMCFG    418
XP_006931832.1      SLVLARILFVPLLLLCNVQPRRYL----AVVFEHDAWFIIFMAAFAFSNGYLASLCMCFG    418
XP_016068296.1      SLVLARLVFVPLLLLCNVHPRRYL----AVAFEHDAWFILFMAAFAFSNGYLASLCMCFG    418
XP_017402910.1      SLVLARLVFVPLLLLCNIKPRRYL----TVVFEHDAWFISFMAAFAFSNGYLASLCMCFG    427
hENT1               SLVLARLVFVPLLLLCNIKPRRYL----TVVFEHDAWFIFFMAAFAFSNGYLASLCMCFG    418
XP_003311378.1      SLVLARLVFVPLLLLCNIKPRRYL----TVVFEHDAWFIFFMAAFAFSNGYLASLCMCFG    444
XP_022813752.1      IANFLRLLLIPWFVLNAAVSNP--------FFTNIVQQCICMATLAFTNGWFNTVPFIVF    363
XP_022713821.1      IGNFLRLLFIPWFVLNAVISSS--------FFTNIVQQCVCIAALAFTNGWFNTVPFIVF    364
EUD71780.1          IGNFLRLLFIPWFVLNAAVSNP--------FFTNIVQQCICIAALGFTNGWFNTVPFMVF    363
SCM11350.1          IGNLLRLLFIPWFVLNASVSSP--------FFTNIVQQCICIAALAFTNGWFNTVPFIVF    363
SCM09553.1          IGNLLRLLFIPWFVLNAAVSNP--------FFTNIVQQCICIAALGFTNGWFNTVPFIVF    363
XP_028534619.1      IANILRVIFVPWFILNAAVDNS--------FFSNTAQQCLCMALFAFTNGWFNTVPFLVF    365
XP_028527991.1      IANAIRIIFIPWFILNSCLKNS--------FFSNIVQHCFCMALFAFTNGWFNTVPFLVF    365
PfENT1-3D7          VANSLRLLFIPWFILNACVDHP--------FFKNIVQQCVCMAMLAFTNGWFNTVPFLVF    365
XP_028862871.1      IANVIRMLFIPWFVLNAAVSKP--------FFESIIQQCICMALLAYTNGWFNTIPFIVF    365
SBT77967.1          IANFLRLLFIPWFVLNAAVSKP--------FFTNIVQQCICMALLAFTNGWFNTVPFIVF    365
SBS85808.1          IANFLRLLFIPWFVLNAAVSKP--------FFTNIVQQCICMALLAFTNGWFNTVPFIVF    366
XP_028544692.1      LFNFLRLLFIPWFVLNAAVEYS--------FFTNIVQQCICMAMLAYTNGWFNTVPFLVF    365
XP_002259877.1      VLNFLRLLFIPWFIINAACEHP--------FFNNIVQQCICMAMLAFTNGWFNTVPFLVF    365
XP_019916700.1      VLNFLRLLFIPWFIMNAACDLS--------IFTNIVQQCICMAMLAFTNGWFNTVPFLVF    365
XP_001614251.1      LLNFLRLLFIPWFVINAACDLP--------IFTNIVQQCVCMAMLAFTNGWFNTVPFLVF    365
                         *: ::* :::                *         :  :. :**:: :: : .
```

```
XP_004935447.1      P----KKVLAHEAETAGAVMAFFLTLGLALGAAISFLFQILI------------------    449
NP_001025348.1      P----KKVSQHEAETAGAIMAFFLSLGLAVGAALSFGFRNMI------------------    440
NP_113872.1         P----KKVKPAEAETAGNIMSFFLCLGLALGAVLSFLLRALV------------------    457
AAH04828.1          P----KKVKPAEAETAGNIMSFFLCLGLALGAVLSFLLRALV------------------    460
XP_003128473.4      P----KKVTPAEAETAGAIMAFFLSLGLALGAVFSFLFRAIV------------------    483
XP_005223466.1      P----KKVKPAEAETAGAIMAFFLSLGLALGAVFSFLFRAIV------------------    456
XP_010861397.1      P----KKVKPAEAETAGAIMAFFLSLGLALGAVFSFLFRAIV------------------    456
XP_006931832.1      P----KKVKPAEAETAGAIMAFFLSLGLALGAVFSFLFRSIV------------------    456
XP_016068296.1      P----KKVKPAEAETAGAIMAFFLSLGLALGAVFSFLFRAIV------------------    456
XP_017402910.1      P----KKVKPAEAETAGAIMAFFLCLGLALGAVFSFLFRAIV------------------    465
hENT1               P----KKVKPAEAETAGAIMAFFLCLGLALGAVFSFLFRAIV------------------    456
XP_003311378.1      P----KKVKPAEAETAGAIMAFFLCLGLALGAVFSFLFRAIV------------------    482
XP_022813752.1      VKELKKVKHQKDIETISRIMVVSLFFGLFFGMLTTCLYDYFPIVISKNQIV---------    414
XP_022713821.1      VKELKKVKHQKDIETISRIMVVSLFFGLFFGMLTTCLYDYFPIGILNN------------    412
EUD71780.1          VKELKKVKSQKDIEVISRIMVLALFCGLFFGMLTTCIYDQFPIVIPKPT-----------    412
SCM11350.1          VNELKTVKNQKDVEVISRIMVLALFCGLFFGMLTTCLYDYFPIVIEAAAKK---------    414
SCM09553.1          VNELKKVKNQKDIEVISRIMVLALFCGLFFGMLTTCLYDKFPIVIKAVAK----------    413
XP_028534619.1      VQELKKAKKPKDIETISTFMVIAMFVGLFSGIWTTYIYDLFPIVN---------------    410
XP_028527991.1      VNELKVAKKPKDIETVSTFMVIAMFLGLCAGIWSTYIYDLFPIVIKM-------------    412
PfENT1-3D7          VKELKKAKKKKEIEIISTFLVIAMFVGLFCGIWTTYIYNLFNIVLPKPDLPPIDVTQ---    422
XP_028862871.1      VQELKKAKKKKDIETISTFMVIAMFVGLFMGIWTTYIYNLFPLVPESKAEL---------    416
SBT77967.1          VQELKKAKKKKDIETISTFMVVAMFVGLFMGIWTTYIYDLFPIVIKKQVPL---------    416
SBS85808.1          VQELKKAKKKKDIETISTFMVVAMFVGLFMGIWTTYIYDLFPIVIKKQVPVALNSADSLG    426
XP_028544692.1      VQELKKTKKKKDIETISTFLVIAMFVGLGMGIWTTYIYNYFPIVIKRYVIP---------    416
XP_002259877.1      VQELKKAKKKKDIETIATLLVVAMFVGLFMGIWTTYIYDLFPIVIERPVVP---------    416
XP_019916700.1      VQELKKAKKKKDIETISTFLVVAMFVGLFMGIWTTYIYDYFPIVIKRPVVP---------    416
XP_001614251.1      VQELKKAKKKKDIETISTFLVVAMFVGLFMGIWTTYIYDFFPIVIKRYVVP---------    416
                               : *  . .: . :  **  *   :     :

XP_004935447.1      ---------  449
NP_001025348.1      ---------  440
NP_113872.1         ---------  457
AAH04828.1          ---------  460
XP_003128473.4      ---------  483
XP_005223466.1      ---------  456
XP_010861397.1      ---------  456
XP_006931832.1      ---------  456
XP_016068296.1      ---------  456
XP_017402910.1      ---------  465
hENT1               ---------  456
XP_003311378.1      ---------  482
XP_022813752.1      ---------  414
XP_022713821.1      ---------  412
EUD71780.1          ---------  412
SCM11350.1          ---------  414
SCM09553.1          ---------  413
XP_028534619.1      ---------  410
XP_028527991.1      ---------  412
PfENT1-3D7          ---------  422
XP_028862871.1      ---------  416
SBT77967.1          ---------  416
SBS85808.1          RMSQPNKQN  435
XP_028544692.1      ---------  416
XP_002259877.1      ---------  416
XP_019916700.1      ---------  416
XP_001614251.1      ---------  416
```
